# Interpretable Machine‐Learning and Big Data Mining to Predict Gas Diffusivity in Metal‐Organic Frameworks

**DOI:** 10.1002/advs.202301461

**Published:** 2023-05-11

**Authors:** Shuya Guo, Xiaoshan Huang, Yizhen Situ, Qiuhong Huang, Kexin Guan, Jiaxin Huang, Wei Wang, Xiangning Bai, Zili Liu, Yufang Wu, Zhiwei Qiao

**Affiliations:** ^1^ Guangzhou Key Laboratory for New Energy and Green Catalysis School of Chemistry and Chemical Engineering Guangzhou University Guangzhou 510006 China; ^2^ Joint Institute of Guangzhou University & Institute of Corrosion Science and Technology Guangzhou University Guangzhou 510006 China

**Keywords:** diffusivity, interpretable machine learning, metal‐organic frameworks, polarizability, selectivity

## Abstract

For gas separation and catalysis by metal‐organic frameworks (MOFs), gas diffusion has a substantial impact on the process' overall rate, so it is necessary to determine the molecular diffusion behavior within the MOFs. In this study, an interpretable machine learing (ML) model, light gradient boosting machine (LGBM), is trained to predict the molecular diffusivity and selectivity of 9 gases (Kr, Xe, CH_4_, N_2_, H_2_S, O_2_, CO_2_, H_2_, and He). For these 9 gases, LGBM displays high accuracy (average *R^2^* = 0.962) and superior extrapolation for the diffusivity of C_2_H_6_. And this model calculation is five orders of magnitude faster than molecular dynamics (MD) simulations. Subsequently, using the trained LGBM model, an interactive desktop application is developed that can help researchers quickly and accurately calculate the diffusion of molecules in porous crystal materials. Finally, the authors find the difference in the molecular polarizability (*ΔPol*) is the key factor governing the diffusion selectivity by combining the trained LGBM model with the Shapley additive explanation (SHAP). By the calculation of interpretable ML, the optimal MOFs are selected for separating binary gas mixtures and CO_2_ methanation. This work provides a new direction for exploring the structure‐property relationships of MOFs and realizing the rapid calculation of molecular diffusivity.

## Graphical abstract

In this study, the rapid calculation of molecular diffusivity in metal‐organic frameworks by training the LightGBM model is realized, and the difference in the molecular polarizability (*ΔPol*) is found to be the key factor governing the diffusion selectivity by using Shapley additive explanation.

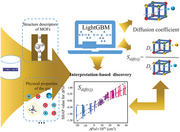

## 1 Introduction

Metal‐organic frameworks (MOFs) are a class of crystalline materials composed of metal ions or clusters and organic linkers.^[^ ^1^ ^]^ Compared with traditional porous materials such as activated carbon and zeolite, which consist of relatively rigid inorganic building units, MOFs have the characteristics of high specific surface area, high porosity, adjustable pore size, highly predictable variable structure, and chemical diversity, making them have great potential in gas adsorption,^[^ ^2^ ^]^ separation,^[^ ^3^, ^4^ ^]^ and catalysis.^[^ ^5^, ^6^ ^]^ For many applications, gas diffusivity is important. For example, slow diffusion rates will limit the dynamics of adsorption process, thus affecting the adsorption performance.^[^ ^7^ ^]^ However, in kinetic or membrane separation, the diffusivity difference of different molecules can be used to achieve gas separation.^[^ ^8^ ^]^ For instance, owing to the different diffusion coefficients, propylene and propane can be separated by Zeolitic Imidazolate Framework‐8 (ZIF‐8)^[^ ^9^ ^]^ and Zr‐fum‐fcu‐MOF.^[^ ^10^ ^]^ Additionally, in the field of catalysis, using a catalyst with high reactant diffusivity can improve the catalytic efficiency.^[^ ^5^, ^6^ ^]^ Liu and coworkers reported the construction of an intriguing hollow mesoporous MOF nanostructure, which enhanced the diffusion of 4‐chlorostyrene and improved the catalytic efficiency for 4‐chlorostyrene oxidation.^[^ ^5^ ^]^ MOFs are promising candidates for membrane separations, kinetic separations, and catalysis, so it is necessary to understand the molecular diffusion in MOFs.

Depending on the driving force, there are two main types of diffusion: transport diffusion and self‐diffusion. For dilute loadings, the self‐diffusion coefficient and the transport diffusion coefficient are similar.^[^ ^11^, ^12^, ^13^ ^]^ Traditional experimental techniques for measuring self‐diffusion coefficient include pulsed‐field gradient PFG NMR,^[^ ^14^, ^15^ ^]^ quasi‐elastic neutron scattering (QENS)^[^ ^13^ ^]^ and other labeling experiments.^[^ ^16^ ^]^ Over the last 10 years, numerous MOFs have been designed, synthesized, and predicted. Based on whether the MOF is obtained experimentally or predicted theoretically, two MOFs databases have been established by Snurr group: the Computation‐Ready Experimental MOFs (CoRE‐MOFs) database and^[^ ^17^, ^18^ ^]^ the hypothetical MOFs database.^[^ ^19^ ^]^ Considering the large number of MOFs, experimental measurement of the diffusion coefficient is unrealistic. Molecular simulation has been used to study the gas diffusion in MOFs. Chokbunpiam et al.^[^ ^20^ ^]^ investigated the diffusion selectivity of N_2_/NO_2_ mixture in three ZIF materials (ZIF‐8, ZIF‐90, and ZIF‐78) by molecular dynamics (MD) simulation and found that ZIF‐78 had the highest diffusion selectivity. Though molecular simulation techniques can significantly make up for some shortage of experiments, the computational cost is still high, while the efficiency is low due to the huge number of MOF structures. As an alternative, Machine Learning (ML), which can automatically learn the data patterns and optimize the model parameters, has been widely utilized in big data analysis. In ML, the model for training is built using the structure and properties data of the current materials, and machine learning models with high training accuracy have been widely used to study the complex structure‐property relationships and accelerate the discovery of molecules and materials.^[^ ^21^, ^22^, ^23^ ^]^ Ibrahim B. Orhan et al.^[^ ^21^ ^]^ successfully obtained the trained RF models for self‐diffusivities and Henry's constants calculation using the O_2_ and N_2_ uptakes from the CoRE‐MOFs database. The obtained models were then used to predict such properties for a hypothetical MOF data set and to identify structures with a high O_2_/N_2_ selectivity at room temperature. Wu et al.^[^ ^22^ ^]^ constructed multiple ML models to predict the ethane/ethylene separation performance of 425 computationally generated defective UiO‐66 structures. And according to the feature importance analysis, it is determined that the missing linker ratio has significant influence on the target characteristics, while the missing linker SRO descriptor has a negligible impact. However, determining the quantitative structure‐property relationships is challenging, even if it is not difficult to determine that variables are significant qualitative indicators from machine learning models' built‐in factor importance weights. To solve this problem, based on the molecular simulation data, we build a light gradient boosting machine (LGBM) model with high precision, high stability and high computational efficiency, and use the Shapley Additive Explanations (SHAP) technique to big data mine and quantify the complex structure‐property relationships. SHAP is a popular method for interpreting model predictions by calculating the contribution of each input feature.^[^ ^23^, ^24^ ^]^ This technique uses game theory to enhance the analysis of the relative importance of each variable in machine learning prediction, and has been extensively utilized to analyze the machine learning models in many research fields including materials and drugs development.^[^ ^25^, ^26^, ^27^, ^28^ ^]^ For example, Zhou et al.^[^ ^25^ ^]^ utilized interpretable ML approaches to screen out high performance MOFs for the separation of ethane and ethylene through obtaining structure characteristics, and identifying and validating promising MOFs from the structure features. Despite having high accuracy in predicting MOF performance, these models are still somewhat limited and are only appropriate for specific single separation systems.

We aim to build a more representative model based on results from molecular dynamics simulations and employ the SAHP technique to mine the physical descriptors of MOFs as well as the structure‐activity relationship between the physical properties and the diffusion properties of MOFs. In the second section, the atomic models of CoRE‐MOFs and gas molecules, simulation method, ML principle, and calculation method of SHAP value are introduced. In the third section, the structure‐activity relationship between the properties of the model, the diffusion properties of MOFs, and the characteristic descriptors are discussed in detail, and the best MOF materials in catalysis and separation are determined. In addition, an interactive desktop application is designed for computing of molecular diffusivity. Finally, some unexpected and important findings from the study are summarized and analyzed.

## 2 Methods

### 2.1 Molecular Model

In this work, a computational screen was performed for the crystal structure of CoRE‐MOFs from the database established by Chung et al.^[^ ^18^ ^]^ The structure parameters of CoRE‐MOFs were derived from experimental data after the free solvent molecules were removed. The Lennard‐Jones (LJ) and the electrostatic potentials were used to describe the atomic frameworks of the MOFs:

(1) $$\sum 4\varepsilon_{\mathrm{ij}}\left[{\left(\frac{\sigma_{\mathrm{ij}}}{r_{\mathrm{ij}}}\right)}^{12}-{\left(\frac{\sigma_{\mathrm{ij}}}{r_{\mathrm{ij}}}\right)}^{6}\right]+\sum \frac{q_{i}q_{j}}{4\pi \varepsilon_{0}r_{\mathrm{ij}}}$$

where *ε_ij_* represents the potential well depth, *σ_ij_* represents the equilibrium distances, *q_i_*, *q*_j_ represents the atomic charge of atoms *i* and atoms *j*, respectively, and *ε_0_* = 8.8542×10^−12^ C^2^ N^−1^, which is the permittivity of vacuum. The LJ potential parameters of the MOFs were taken from the universal force field (UFF),^[^ ^29^ ^]^ and they are listed in Table S1 (Supporting Information). The atomic charge of MOFs was calculated by the method of MOF electrostatic potential optimization charge scheme (MEPO‐QEq).^[^ ^30^ ^]^ Previous work^[^ ^31^ ^]^ shows that the adsorption and diffusion performance of MOFs can be predicted appropriately using the UFF force field. Furthermore, five structure descriptions of MOF were calculated, including pore limiting diameter (PLD, Å), large cavity diameter (LCD, Å), volumetric surface area (VSA, m^2^ cm^−3^), void fraction (*ϕ*), and density (*ρ*, kg m^−3^). The PLD and LCD were calculated in the Zeo++ package.^[^ ^32^ ^]^ The VSA and *ϕ* were calculated under RASPA software package using N_2_ with a diameter of 3.64 Å and He with a diameter of 2.58 Å as probes, respectively.^[^ ^33^ ^]^

We constructed ten gas components (C_2_H_6_, Xe, CH_4_, Kr, N_2_, H_2_S, O_2_, CO_2_, H_2_, and He) and the force field parameters of the gases are shown in Table S2 (Supporting Information), which were derived from the transferable potentials for phase equilibria (TraPPE) force field.^[^ ^34^ ^]^ C_2_H_6_ molecules were described as uncharged united‐atom models with one pseudo‐atom representing –CH_3_ group located at the position of carbon atom. Based on the kinetic diameters, the atomic diameters of Xe and Kr were set as 4.10 and 3.66 Å. CH_4_ was represented by a joint atomic model. N_2_ was represented by a three‐point model, where the bond length of N—N was 1.10 Å. H_2_S was represented by a four‐bit model with an S—H bond length of 1.13 Å and LJ potential energies applied on the S and H atoms. Furthermore, a virtual atom was located near the S atom.^[^ ^35^ ^]^ O_2_ was represented by a three‐point model. For CO_2_, the bond length of C—O was 1.16 Å, and ∠OCO was 180°. The atomic diameter of He was 2.58 Å.

### 2.2 Molecular Simulation

To evaluate the diffusivity (*D*) and diffusion selectivity (*S*_diff_) of MOFs, the diffusion coefficients of C_2_H_6_, Xe, CH_4_, Kr, N_2_, H_2_S, O_2_, CO_2_, H_2,_ and He in CoRE‐MOFs under 298 K and 1 bar were calculated by MD simulation. Each MOF ran an MD simulation individually. The cross‐interaction between MOFs and adsorbed molecules was calculated by the Lorentz‐Berthelot rule, and the time step of the MD simulation was 1 fs. In each simulation, the MOF atoms were assumed to be rigid with atoms frozen. The periodic boundary was applied to a 3D system to simulate cells with a 3D direction of at least 24 Å. A long‐range correction was included in the LJ interaction calculation with a spherical cutoff of 12.0 Å. The electrostatic interaction between the frame and the gas molecule was calculated by the Ewald sum method. The MD duration for each MOF was 5 ns, with the last 2 ns used for production. All MD simulations were performed using the Raspa package.^[^ ^33^ ^]^ To further verify the accuracy of the simulation, we compared the simulated values of the molecular diffusion coefficient with the experimental values. As shown in Figure S1a (Supporting Information), most of the data were distributed on the diagonal, indicating that our molecular simulation values have a similar tendency to the experimental values. Additionally, the reliability of our method has also been verified in many studies.^[^ ^31^, ^36^ ^]^ To quantify the diffusion separation of two gases, the diffusion selectivity (*S*_diff_) between the ideal binary gas mixtures is estimated by using Equation (2):

(2) $$S_{\mathrm{diff}(i/j)}=\frac{D_{i}}{D_{j}}\;$$

where *D_i_* and *D_j_* are the diffusivities of components *i* and *j* in MOFs, respectively, *i* and *j* are indices that represent C_2_H_6_, Xe, CH_4_, Kr, N_2_, H_2_S, O_2_, CO_2_, H_2,_ and He, and *i/j* represent the different gas mixtures, which are listed in Table S4 (Supporting Information).

### 2.3 Algorithms

In this work, the *D* and *S*_diff_ were predicted by four algorithms. Before machine learning, to make our trained model more universal and able to predict more molecules with different characteristics, we constructed a diverse library including 9 gases with different sizes, shapes, and polarities, and 6013 MOFs from the 2019 CoRE‐MOF database with different topologies and chemical compositions to train the model (as shown Figure S2, Supporting Information). Additionally, we vertically arranged the dataset to make it facilitate the exploration of diffusion similarities among different molecules in MOFs. First, the *D* of nine gas molecules (Xe, CH_4_, Kr, N_2_, H_2_S, O_2_, CO_2_, H_2_, and He) were calculated by molecular dynamics simulation and 54 000 pieces of data were obtained after processing. The Equation (2) was used to calculate the *S*_diff_ of 36 ideal binary gas mixtures, and after processing, 215 846 pieces of data were obtained. Then the machine learning was performed. Numerous studies have shown that the diffusion of gases in MOFs may be affected by atom types, chemical descriptors and structure descriptors of MOF. However, it is shown that the physical features of MOF have significant impact, while atom types and chemical descriptors weakly correlate with gases diffusivity in MOFs.^[^ ^37^, ^38^, ^39^ ^]^ Therefore, we selected the physical features of MOFs as descriptors. In addition, considering the diffusion of gas molecules in the MOF may also be affected by the gas physical properties,^[^ ^8^, ^40^ ^]^ we simultaneously added the physical properties of gas molecules as descriptors to predict gases diffusion in MOFs. The characteristic variable of target value 1 (*D*) is composed of the structure descriptors of MOF (PLD, LCD, VSA, *ϕ*, and *ρ*) and the physical properties of the gas (Kinetic diameter (*Dia*), Quadrupole moment (*Qua*), Polarizability (*Pol*) and Dipole moment (*Dip*), see Table S3, Supporting Information). The characteristic variable of target value 2 (*S*_diff_) is composed of five structure descriptors of MOF (PLD, LCD, VSA, *ϕ*, and *ρ*), the physical properties of the separated gas (*Dia_i_*, *Qua_i_*, *Pol_i,_* and *Dip_i_*), and the differences in the physical properties of separated gas (*ΔDia*, *ΔPol*, *ΔDi*p, and *ΔQua*),^[^ ^41^ ^]^ which are listed in Table S4 (Supporting Information).

After preprocessing the above data, four ML algorithms, including random forest (RF),^[^ ^42^ ^]^ gradient boosted regression tree (GBRT),^[^ ^43^ ^]^ extreme gradient boosting (XGBoost),^[^ ^44^ ^]^ and LGBM,^[^ ^45^ ^]^ were used to predict *D* and *S_diff_*. For more information on how the ML algorithms work, see Section S3 in the Supporting Information. These four algorithms are all ensemble algorithms, which can solve the inherent drawbacks of a single model or a model with a certain set of parameters, integrate more models and avoid limitations so as to improve the generalizability and robustness of individual models. RF uses randomly selected split data sets to construct decision trees. In RF, the number of trees was 250, the maximum depth of the trees was 14, and the criterion was set as “squared_error”. GBRT is an iterative decision tree algorithm. In GBRT, the loss function was set as “squared”, the maximum number of spanning trees (also the maximum number of iterations) was 400, the learning rate was 0.1, the maximum depth was 10, and the criterion for the split was set as “feldmann_mas”. XGBoost introduces an improvement on boosting algorithms based on gradient boosting decision tree (GBDT). In XGBoost, the maximum number of trees was 520, the maximum depth of the tree was 11, and the learning rate was 0.1. In order to prevent the model from overfitting, the weight of L2 regular term was set to 1.0, and the weight of the L1 Regex was set to 0.8. LGBM is a Histogram‐based decision tree algorithm, which only computes the information gain of high‐gradient data and uses Exclusive Feature Bundling (EFB) to extract features for dimensionality reduction. When building the model, the maximum number of spanning trees was set as 620, the maximum number of leaves of basic learners was set as 400, the colsample ratio of constructing each tree was set as 0.8, and the learning rate was set as 0.1. ML algorithm parameters are detailed in Table S6 (Supporting Information).

ML models are built by training datasets with target attributes and pre‐processing features. Before training, the data set was randomly divided into a training set and a test set in a ratio of 7:3 and then standardized. During training, the k‐fold cross‐validation (CV) method (k = 10 in this work) was adopted to verify the accuracy and stability of the model (as shown in Figure S8, Supporting Information).^[^ ^46^, ^47^, ^48^, ^49^ ^]^ The correlation coefficient *R^2^* and root mean square error (RMSE) were used as the indexes to evaluate the model. All ML models were trained using the scikit‐learn Python package^[^ ^50^ ^]^ (all versions of the tool packages used during training are given in Table S5, Supporting Information). We reiterate that there is no shared MOF between the training set and the test set during any model training.

In this work, Shapley Additive explanations (SHAP) is combined with a trained model to explain the importance and role of different predictors in the analysis. This strategy is based on game theory.^[^ ^24^ ^]^ To calculate SHAP values based on a tree model, we utilize an algorithm called TreeExplainer.^[^ ^23^ ^]^ The prediction is made for each sample in the data set, and then the results of all predictions are plotted to show the global interpretation. Compared with the default relative importance calculation method of the model, SHAP technology can quantify the impact of features on the output of the model from both magnitude (significant or insignificant) and direction (positive or negative) aspects. Additionally, pairwise interactive SHAP interaction values of any two features, which have properties similar to those of SHAP values,^[^ ^51^ ^]^ can be used to further understand the local contribution of features. This allows the interaction effects of individual model predictions to be considered separately. Details of the computation can be found in Section S5 in the Supporting Information. The Python library for calculating SHAP values in this study is available from https://github.com/slundberg/shap, developed by Lundberg et al.^[^ ^23^ ^]^

## 3 Result and Discussion

### 3.1 Univariate Analysis

**Figure 1:**
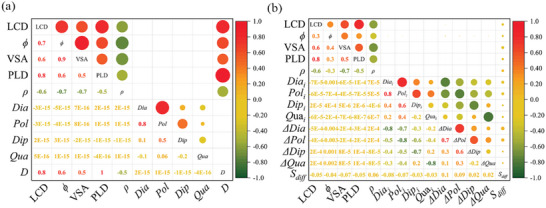
Pearson correlation coefficient matrix of features of CoRE‐MOFs, demonstrating the level of linear correlation between pairwise features. Coefficients +1, −1, and 0 indicate total positive, totally negative, and no linear correlation, respectively. a) on *D*, b) on *S*_diff_.

To explore the internal relations between the descriptors of CoRE‐MOFs, the properties of diffused gases, and the diffusion performance, the correlation between them was calculated (calculation details in Section S2, Supporting Information), as shown in **Figure** **1**a. PLD and LCD, and *ϕ* and VSA are highly positive correlated descriptors and their Pearson correlation coefficients were 0.8 and 0.9, respectively. This is because the PLD and LCD are descriptors of the pore diameter, and VSA and *ϕ* are descriptors of the pore volume. *ρ* depends on both the mass and the pore size of the material, so it has a negative correlation with other geometric descriptors. However, there is a weak and negative association between geometric descriptors and *S*_diff_, which may be due to the wide range of feature values. According to work by Le et al.,^[^ ^21^, ^52^ ^]^ there should be a strong correlation between the structure descriptors and the diffusion selectivity. Therefore, we put some constraints on the descriptors to improve their correlation in the next section. Furthermore, Figure 1b shows a negative correlation between the physical characteristics of gas component *i* (*Dia_i_*, *Qua_i_*, *Pol_i,_* and *Dip_i_*, where *i* represents the gas with smaller dynamic diameter of the *i*, *j* gas mixtures) and diffusion selectivity, which means large *Dia_i_*, *Qua_i_*, *Dip_i_*, and *Pol_i_* values are not favorable for self‐separation. Additionally, as shown in Figure 1b, there is a positive correlation between the difference of physical properties (*j‐i*) of ideal binary separation system (*i/j*) and the *S*_diff_, with the order of *ΔDia* > *ΔPol* > *ΔDip* ≈ *ΔQua*. The use of ML prediction and SHAP value assessment is significantly effective in big data mining and quantifying the relationship between the diffusivity of diffused gases and the material structure. This approach is robust to multicollinearities and can avoid the bias of the linear correlation coefficient, as has been proven by multiple studies.^[^ ^27^, ^28^, ^53^ ^]^

### 3.2 ML Model and Evaluation

**Figure 2:**
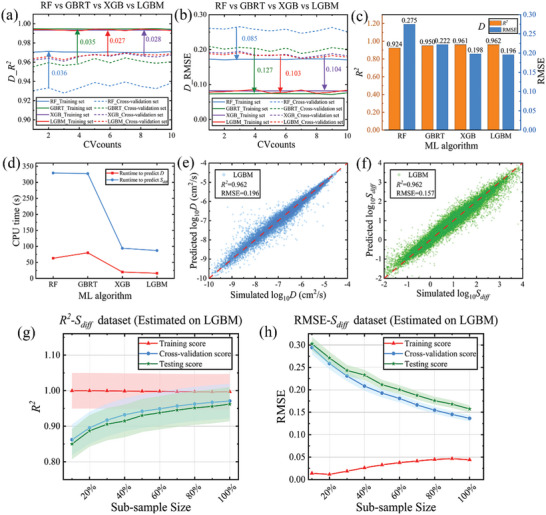
Performance a) *R* ^2^ and b) RMSE comparison of four ML algorithms for predicting *D* in 10‐fold cross‐validation. c) The performance of the four ML algorithms with *D* as the target value on the test set on the model. d) Comparison between the four ML algorithms in terms of computing time. Comparison of the predicted results of e) *D* and f) *S*_diff_ of LGBM algorithm and the simulated results of CoRE‐MOFs on the test set. g) *R^2^* as a function of the sample‐size percentage used for training LGBM model, h) RMSE as a function of the sample‐size percentage used for training LGBM model. The line in the graph shows the mean of 10‐fold cross‐validation. The shaded portion of each line in (g) and (h) represents the error band (±0.05).

Different ML algorithms may be appropriate for various prediction targets and data features. Because previous researches have shown that the tree model performs well in predicting material properties,^[^ ^7^, ^21^, ^52^, ^54^ ^]^ in this work, four ML‐tree model integration algorithms are used to predict the *D* and *S*_diff_, including RF, GBRT, XGBoost, and LGBM. First, the stability and fitting ability of the four models were evaluated. **Figure** **2**a,b and Figure S9a,b (Supporting Information) show the predictive performance of *D* and *S*_diff_ using four ML algorithms and 10‐fold cross‐validation. Compared with the other three algorithms, LGBM has the highest fitting ability and stability (average *R^2^* = 0.967). The *R^2^* and RMSE of the Cross‐validation set and Training set are very close, with the difference of only 0.027 and 0.103, respectively (the cross‐validation results are listed in Table S7, Supporting Information). And it consumed only one‐fifth of GBRT's CPU time (processing 90000 pieces of data in ≈16 s). The second‐place finisher, XGBoost, used a little bit more than LGBM consumption of computation time. Furthermore, Figure 2c and Figure S11d (Supporting Information) present a comparison of the predictive power of *D* and *S*_diff_ across various models on the test set to evaluate the generalizability of the four machine learning models. Results show that LGBM has the best prediction accuracy for *D* (also has the same accuracy for predictions of *S*_diff_, as shown in Figure 2f; Figure S11a–c, Supporting Information). Based on the performance indicators, as shown in Figure 2e,f, the LGBM regression algorithm was selected for this study because of its low cost of running time, good generalization, and high accuracy (*R^2^* = 0.962), and this method has been widely used in many fields.^[^ ^45^, ^55^, ^56^ ^]^ The excellent performance of LGBM may be attributed to its different tree structure from other tree model algorithms, such as GBDT and XGBoost. LGBM uses a leaf‐by‐leaf tree growth strategy to identify the “Best” leaves with the highest gain, splitting only the best leaves, resulting in tree asymmetry. In contrast, the other Gradient boosting variants grow at the tree level, which means that each node in the same level is split into child nodes and undoubtedly increases computational energy consumption. Compared to trees grown horizontally, tree structures built from leaf‐oriented growth may better capture the relationship between MOF descriptions and performance at the same cost.

For the optimal algorithm LGBM, to avoid the effect of training samples on the molecular coverage in the test set, we randomly selected 10, 20, 30…100% of the sub‐samples (a subset of the MOF) for benchmarking. As shown in Figure 2g,h, for both *R^2^* and RMSE metrics of *D*, the accuracy of the Cross‐validation set and Testing set predictions in the LGBM model increases as the sample size grows, while RMSE decreases with increasing sample size. The LGBM training score is much higher for small data size samples than the validation score. When adding more data to the training sample, the generalization capacity of the model will be improved, and the over‐fitting of training samples will be decreased. The same results are observed for the diffusion selectivity prediction. (see Figure S13, Supporting Information). Surprisingly, the accuracy of our selected LGBM model is still very high in a small sample size (*R^2^* = 0.905, under the 10% sample size of testing score), indicating that the LGBM model still has high accuracy even when the algorithm focuses on a small range to make predictions (for example, some materials with excellent diffusion performance) and it can be used to predict the diffusivity of new gases or new MOF materials.

### 3.3 Dig Data Mining

**Figure 3:**
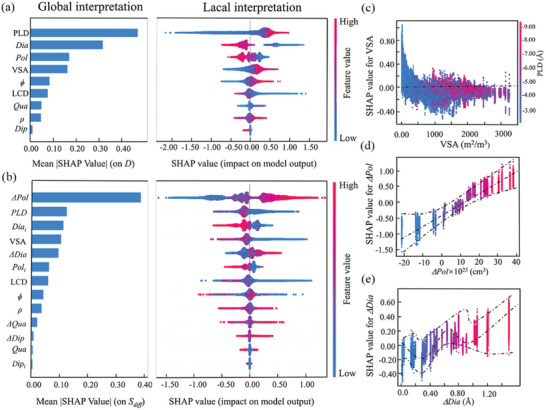
Global feature importance (left, bar charts) and local explanation summary (right, beeswarm plots), in which a dot represents an individual data point in this study, for a) *D* and b) *S*_diff_ values of gases molecule. Note: The dot on the x‐axis shows how each data point impacts the model's prediction for each feature, and when multiple dots are at the same x position, they show the density (Colors represent eigenvalues, where red dots represent high and blue dots represent low; positive SHAP values indicate a positive feature effect on the predicted value, and negative SHAP indicates a negative feature effect on the predicted value). c) SHAP dependence plot of VSA and PLD on making predictions by the LGBM model for *S*_diff_ of gases molecule. d) SHAP main effect of *ΔPol* on *S*_diff_, the dotted line represents the trend of the SHAP value for *ΔPol* with *ΔPol*. e) SHAP main effect of *ΔDia* on *S*_diff_, the dotted line represents the trend of the SHAP value for *ΔDia* with *ΔDia*. The colors in (d) and (e) show the values of *ΔPol* and *ΔDia*, respectively, with red dots represent high and blue dots represent low.

To further explore how the variables affect diffusion performance, the TreeExplainer in SHAP was utilized to explain and mine the relationships between MOF descriptors and gas characteristics.^[^ ^23^ ^]^ In a SHAP module, all features are considered contributors. SHAP values are calculated to quantify the effect of characteristics on performance in terms of magnitude (marked or not marked) and direction (positive or negative). **Figure** **3**a,b display the SHAP values based on the LGBM method, and the bar graph on the left represents global importance (degree of influence on model output), which is the average SHAP absolute value for all points in the dataset (listed in Tables S8 and S9 in the Supporting Information). The beeswarm plots on the right side of Figure 3a,b show the distribution of SHAP values for all MOFs, and analysis for *D* reveals that PLD had the most significant impact on the gas molecule diffusivity in MOFs, which is consistent with previous findings.^[^ ^57^, ^58^, ^59^ ^]^ In Figure 3a, it can be seen that smaller PLD, VSA, and *ϕ* are negatively correlated with diffusivity because diffusion cannot occur when the pore size of a MOF is too small to allow molecules to pass through. According to the previous analysis, VSA, *ϕ*, and PLD are highly correlated, so their effects on diffusion performance are similar. The density of MOF has no direct quantitative correlation with diffusivity. And regarding the characteristics of the gas itself, Figure 3a shows a negative correlation between diffusion performance and gas molecules with a large kinetic diameter and high polarizability, demonstrating that a large molecular kinetic diameter or polarizability will hinder its diffusion. Because not all molecules have quadrupole moments and dipole distances, SHAP values of the *Qua* and *Dip* are concentrated ≈0, indicating that the *Qua* and *Dip* have little effect on the diffusion properties of most molecules, except for certain kinds of gases, such as CO_2_, N_2_, H_2_S.

**Figure 4:**
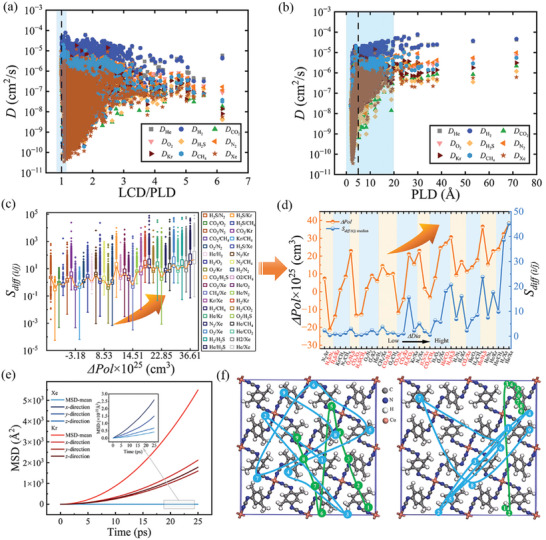
Structure–performance relationships of a) LCD/PLD and *D* and b) PLD and *D*. c) Box and whisker diagrams for the heat of *S*_diff_ of each binary gas mixtures in 6013 MOFs. The center line of each box represents the median of data and the square symbol is centered on the mean of data. The upper and lower horizontal lines provide the minimum value (Q1‐1.5×IQR) and maximum value (Q3+1.5×IQR), respectively, where IQR equals Q3‐Q1. Outliers beyond this range are shown as individual data points. d) Double y‐axis line chart, the y‐axis on the left represents *ΔPol*, and the y‐axis on the right represents the median *S*_diff_ of the 6013 MOFs, the x‐axis is the *ΔDia* of 36 molecular mixtures. e) Total MSDs and the x‐, y‐, and z‐components of MSDs of Kr and Xe in FAPYEA. f) Diffusion pathways of two Xe and Kr molecules in FAPYEA (Xe, green; Kr, blue).

Furthermore, in contrast to the previous trend for PLD, there is a positive correlation between diffusivity and small LCD. This is because when LCD becomes smaller, the PLD to LCD ratio becomes lower, indicating the uniformity of the aperture. As seen in **Figure** **4**a, the diffusion rate is the highest when the ratio of LCD/PLD is close to 1, and then it begins to decline as LCD/PLD values increase. The results demonstrate that diffusion benefits from more uniform pores, which is consistent with previous findings.^[^ ^47^, ^60^ ^]^ To further understand how pore and gas parameters impact the effectiveness of diffusion, we explored the relationship between diffusion coefficients of the nine distinct gases and PLD under real data, as seen in Figure 4b. It is found that when PLD increases, *D* increases rapidly and then stabilizes gradually. However, there is still a significant difference between the ultimate trend of *D* for various gas characteristics and the rising rate. Consequently, the properties of material, such as separation and diffusion‐dominated catalytic performance, may be significantly improved by combining PLD and gas properties (kinetic diameter and polarity).

In some fields, such as gas separation, the diffusion selectivity is also a very important index, so we also analyzed the diffusion selectivity. As shown in the top‐left corner of Figure 3c and Figure S14 (Supporting Information) (blue dots), the SHAP value is benefited by small LCD, *ϕ*, VSA, which is higher than zero, indicating a positive effect. More intriguingly, when the PLD is in the range of 2.4–5.0 Å, the LCD is in the range of smaller than 10 Å, the *ϕ* is ≈0.1, and the VSA is ≈500 m^2^ cm^−3^, and there will be a peak of SHAP value higher than zero, which will have a positive impact on the target value of our ML model. In other words, MOF in this range exhibits high diffusion selectivity. And regarding the characteristics of the gas itself, the kinetic diameter of a gas molecule (*Dia*) or the difference between the kinetic diameters of two guest molecules (*ΔDia*) is the most significant factor affecting the diffusion selectivity, which has been mentioned in many studies.^[^ ^61^, ^62^ ^]^ However, Figure 3b indicates that *ΔPol* is the main factor influencing the diffusion selectivity, and its influence is even stronger than *ΔDia*. Some studies suggest that the polarization difference can also affect the diffusion selectivity.^[^ ^8^, ^61^ ^]^ Then the influence of *ΔPol* on diffusion selectivity was quantified using diffusion data mining of 36 binary gas mixtures. The results of SHAP analysis show that the influence of *ΔPol* on diffusion selectivity of gas mixtures was much greater than that of *ΔDia*. From Figure 3d,e, we find that although *S*_diff_ increases with the increase of *ΔPol* and *ΔDia*, the relationship between *S*_diff_ and *ΔPol* is linear, whereas the relationship between *S*_diff_ and *ΔDia* is partially downward. To verify the rule of the big data mining and SHAP results, we plotted the data distribution of diffusion selectivity of 36 binary gas mixtures (represented by the median of *S*_diff(i/j)_) under real data, as shown in Figure 4c,d. Although *S*_diff(i/j)_ increases as *ΔDia* increases, there are many obvious deviations, as shown in the orange area in Figure 4d. When analyzing the reasons for the deviations, we found that the *ΔDia* of H_2_S/N_2_ and N_2_/Kr is similar, but the *S*_diff_ of N_2_/Kr is five times that of H_2_S/N_2_, this is because the *ΔPol* of H_2_S/N_2_ (*ΔPol* = 7.441×10^−25^ cm^3^) is much larger than that of H_2_S/N_2_ (*ΔPol* = ‐21.257×10^−25^ cm^3^) (see Figure 4d). Therefore, *ΔPol* plays a dominant role. Meanwhile, it is worth noting that H_2_S has a dipole moment and CO_2_ has a quadrupole moment, so they are more prone to polarization than other molecules and have a better affinity with MOF, making their diffusion coefficients decrease, resulting in the specific *S*_diff_ sequence of H_2_S and CO_2_ mixtures. For the third orange region in Figure 4d, the *S*_diff_ of the four gas molecular mixtures follows the sequence: *S*_diff(CO2/N2)_ < *S*_diff(CO2/H2S)_ < *S*_diff(O2/CH4)_ < *S*_diff(CH4/Xe)_, which is consistent with the sequence of *ΔPol*: *ΔPol*_(CO2/N2)_ (−11.707×10^−25^ cm^3^) < *ΔPol*_(CO2/H2S)_ (9.55×10^−25^ cm^3^) < *ΔPol*_(O2/CH4)_ (10.118×10^−25^ cm^3^) < *ΔPol*_(CH4/Xe)_ (14.51×10^−25^ cm^3^), demonstrating that the trend of *S*_diff_ is the same as that of *ΔPol*. The comparison of the orange and blue lines in Figure 4d and Figure S15b (Supporting Information) clearly shows that the trend of *S*_diff_ is more consistent with the trend of *ΔPol*, especially in the offsets in the orange areas. These results illustrate that *ΔPol* is the most important factor for *S*_diff_. Figure 4d also shows that *S*_diff_ and *ΔPol* are monotonically increasing, which is consistent with the interpretation of characteristics using SHAP. Therefore, big data mining using SHAP can accelerate the discovery of the structure‐property relationship of molecular diffusion in MOFs and provide insights for the design of nano‐porous materials in gas separation.

Then we studied the mechanism of *ΔPol* affecting diffusion selectivity. For example, in the separation of inert gases Kr/Xe, the diffusion trajectories of Kr and Xe in various MOFs were explored. As shown in Figure 4e and Figure S16 (Supporting Information), Xe has a higher mean squared displacement (MSD) than Kr at any time (diffusivity can be measured directly from MD trajectories using MSD, see Section S5, Supporting Information). Figure 4f shows that the Xe molecular is routinely diffused along the same path, and the Xe molecular diffusion trajectories are all narrower than those of Kr. This phenomenon was also seen in other MOFs (see Figure S17, Supporting Information). We speculate that this may be because Xe and Kr have quite different polarizabilities, with Xe (40.44×10^25^ cm^3^) having a polarization that is nearly twice that of Kr (24.84×10^25^ cm^3^). MD simulation results confirm that the interaction between Xe and MOFs is stronger than the interaction between Kr and MOF, as shown in Table S10 (Supporting Information), owing to the higher polarizability of Xe.^[^ ^63^ ^]^ This means that the molecule with high polarizability (Xe) has a better affinity with MOF,^[^ ^64^ ^]^ making it adsorbed preferentially and diffuse slower, thus increasing its selectivity over the molecules with lower polarizability (Kr). However, the CO_2_‐participating gas mixtures deviates from the regular pattern (for example, CO_2_/Xe, H_2_/CO_2_). On the one hand, CO_2_ has a larger quadrupole moment than other molecules, resulting in strong Coulombic interactions with MOFs, which hinders diffusion.^[^ ^65^, ^66^ ^]^ On the other hand, 64.5% of the MOFs used in this work have open metal sites, and CO_2_ tends to polarize at the open metal sites,^[^ ^67^ ^]^ which enhances the affinity between CO_2_ and MOF, leading to decrease of *D* and affects the gas diffusion selectivity. Therefore, *ΔPol* affects the diffusion of gas molecules mainly through the interaction force, while *ΔDia* affects the diffusion through the steric hindrance effect. Through the big data mining in this work, we find that *ΔPol*, which affects the interaction force, plays a leading role in the diffusion selectivity of the gas mixtures, and accompanied by *ΔDia*, which is the second most important factor, it can effectively predict the gas diffusion behavior in MOFs and design new materials with high selectivity.

### 3.4 Application

#### 3.4.1 Prediction of Diffusivity of Other Gases (Extrapolation)

**Figure 5:**
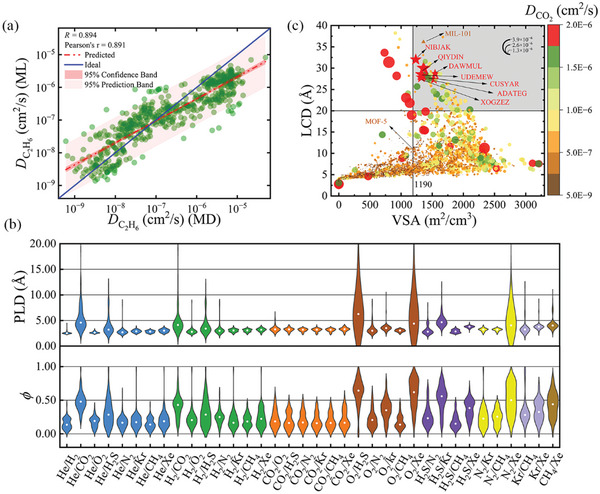
a) Predicted results of *D* by the final LGBM model versus MD‐calculated values in the 534 test set MOFs. b) Approximate distribution of PLD and *ϕ* of about TOP 100 MOFs for 36 mixed gas mixtures. The colors represent different gas mixtures, and the white ball represents the median.c) The LCD versus VSA diagram, the size of the point represents the value of *D*_CO2_, and the larger the point, the bigger the value of *D*_CO2_.

The big data mining of gas diffusion performance in this work is promising and it can anticipate and extrapolate the diffusion behavior of other gases. As shown in the previous sections, our trained LGBM model can predict the *S*_diff_ of 36 mixtures of ideal binary gas as well as the diffusivity of nine gas molecules in MOFs with reasonable accuracy, making it possible to predict the diffusion behavior of more gases, with lower calculation cost. Except for the nine gases, the diffusion of C_2_H_6_ with higher polarizability was also predicted using our LGBM models by employing descriptors for C_2_H_6_ and MOF. The best MOF was screened by ML prediction, and then molecular simulation computation was performed to confirm the logic of the LGBM extrapolation approach. After finishing the prediction of diffusion selectivity, diffusion selectivity thresholds for eight binary gases containing C_2_H_6_ (see Table S11, Supporting Information) were determined and the Top 100 MOFs were chosen. Figure S18 (Supporting Information) shows the best range of five structure descriptors for the TOP 100 MOFs for eight ideal binary gas mixtures separations, which are like the structure‐activity relationships of the 36 gas mixtures computed by high throughput calculation. After removing overlapping MOFs (that is, MOFs with excellent performance in various separation systems), 534 MOFs with the best C_2_H_6_ diffusion performance were chosen using the LGBM algorithm. 534 optimum MOFs were subjected to MD simulations, which had an *R*‐value of up to 0.894 when compared to ML predictions, confirming the accuracy of ML (as shown in **Figure** **5**a). The outcomes of molecular simulation and ML for *D*_C2H6_ prediction demonstrate the feasibility of the extrapolation strategy. As shown in Figure 5a, a definite positive correlation exists between the ML predicted values and the molecular simulated values, but the predicted values are systematically underestimated. Despite the fact that the upper and lower bound ranges are accurately predicted and overpredicted, respectively, MOF over the threshold will be chosen when we apply this model to forecast molecular diffusivity, which is thought to be of practical importance and supported by the study conducted by Borboudakis G et al.^[^ ^68^ ^]^ The diffusion of gas molecules in MOF or other porous crystal materials can be determined individually or in batches by the user with only inputting parameters into an interactive desktop application we develop based on LGBM model (see Figure S19, Supporting Information, the Github repository can be accessed from the link provided in https://github.com/guoshuya1234/Pred_D_software.git). As a result, researchers can predict and assess the diffusion of gas molecules in a material more efficiently.

#### 3.4.2 MOF Screening in the Field of Kinetic Separation and Catalysis

To effectively use the results of AI big data mining to forecast and construct the optimal MOFs for each mixture of binary gas mixtures, we first identified the optimal MOFs by defining thresholds (details are presented in Table S13, Supporting Information) and the Top 100 MOFs for the 36 gas mixtures were selected. The range of structure descriptors (PLD, LCD, *ρ*, LCD/PLD, *ϕ*, VSA) for 36 ideal binary separation systems of about the Top 100 MOFs are shown in Figure 5b and Figure S20 (Supporting Information). The range of PLD and LCD is almost the same, with the range of 2.5–5 Å, *ρ* is in the range of 1000–2500 kg cm^−3^, *ϕ* is in the range of 0–0.3, and VSA is in the range of 0–200 m^2^ cm^−3^, all of which are consistent with the results of the SHAP dependent plot (see Figure 3c; Figure S14, Supporting Information). This exemplifies how the SHAP interaction graph might find the optimum MOF. The optimal TOP 3 MOFs for the separation of different gas mixtures are listed in Table S14 (Supporting Information), which provides some references for gas separation systems based on kinetic differences. Additionally, in the previous section, we discovered that *ΔPol* had a significant influence on *S*_diff_. To increase selectivity and enhance the gas dynamic separation effect, specific polar functional groups or metal particles can be embedded in MOF, which increases the affinity of MOF with strong polar molecules and reduces the diffusion behavior. Many similar studies have been carried out.^[^ ^67^, ^69^, ^70^, ^71^ ^]^ For example, by inserting highly polarized functional groups like bromine into micropores of highly porous materials, Kim et al.^[^ ^71^ ^]^ developed stable MOF materials for CH_4_/N_2_ separation. Therefore, in addition to the pore structure, active sizes with high polarization in the pores should be considered when designing MOF materials with excellent dynamic separation performance.

Beyond gas separation, MOFs has also been widely used in the field of catalysis. For example, recent studies show that MOFs exhibit good catalytic activity for converting CO_2_ to valuable compounds.^[^ ^72^, ^73^, ^74^ ^]^ For gas‐dominated catalytic reactions, it is very important to design catalysts with high gas diffusivity.^[^ ^6^, ^72^, ^75^ ^]^ Lazar et al.^[^ ^76^ ^]^ compared the catalytic activity of microporous Ni@UiO‐66 and mesoporous Ni@MIL‐101 for CO_2_ methanation and found that the mesoporous Ni@MIL‐101 exhibited better catalytic activity than the microporous Ni@UiO‐66 at 280 °C. This is because the diffusion of CO_2_ in mesoporous materials is faster than that in microporous materials, and MOF catalysts with strong CO_2_ diffusivity can make it easier for the reactant to access the catalytic sites and accelerate the catalytic rate, which is consistent with the conclusion of Gao et al.^[^ ^6^ ^]^ In addition, as catalytic supports, MOFs with high specific surface area and high dispersion of active sites (such as MOF‐5 and MIL‐101), usually perform well in the CO_2_ methanation process.^[^ ^76^, ^77^ ^]^ Therefore, MOF catalysts with high specific surface area, high diffusion rate, and mesoporous structure would be a great option for gas‐dominated catalytic reactions. Based on this CO_2_ catalytic reaction, we did a screening for MOFs. According to the results of literature statistics,^[^ ^6^, ^72^, ^76^, ^77^, ^78^ ^]^ the threshold was set as LCD > 20 Å and VSA > 1190 m^2^ cm^3^, with considerable CO_2_, then 65 optimal MOFs were selected (as shown in Figure 5c, and Table S15, Supporting Information). The 58th‐ranked MIL‐101 has been reported to have good catalytic activity toward CO_2_ methanation by Lazar et al.,^[^ ^76^ ^]^ indicating the logic and legitimacy of this screening. Based on gas diffusion properties, we can predict and evaluate high‐performance MOFs catalyst for not only CO_2_ methanation but also other diffusion‐dominated reactions, which is of great significance.

## 4 Conclusion

In this work, the LGBM model exhibits the accurate prediction (*R^2^* = 0.962) for the *D*/*S*_diff_ of nine molecules with varying polarities, sizes, and shapes, after the MD of high‐throughput screening. Meanwhile, the SHAP value is employed to mine the relationships between the prediction components and *D*/*S*_diff_, demonstrating that the PLD is the primary factor governing the molecular diffusivity, and indicating that the molecular diffusivity is severely hampered by the polarizability and kinetic diameter of the molecules, but *ΔPol* dominates the diffusion selectivity of binary gas mixtures. Both the top‐performing MOFs for dynamics separations and for the catalysis of CO_2_ methanation were identified. Based on interpretable ML and big data mining the strategy for designing MOF is established, which provides some theoretical support for the experimental research. Additionally, the trained LGBM model was used to predict and to extrapolate the diffusion of C_2_H_6_. After the comparison between ML results and MD calculations, the simulations were in accordance with the outcomes of ML prediction, indicating that the LGBM extrapolation approach is reasonable and rational. Finally, an interactive desktop application was compiled using the trained LGBM model to assist the user in rapidly gaining molecular diffusion in porous crystal materials. Using datasets from the field of materials science, this work demonstrates the incredible potential of interpretable ML and big data mining to find novel, surprising discoveries. This study reveals the key factors governing gas diffusion, develops interface software for the prediction of diffusivity, and identifies the best MOFs for the applications of gas separation and catalysis from a large collection of MOFs.

## Conflict of Interest

The authors declare no conflict of interest.

## Supporting information

Supporting Information

## Data Availability

The data that support the findings of this study are available in the supplementary material of this article.

## References

[1] A. H. Mashhadzadeh , A. Taghizadeh , M. Taghizadeh , M. T. Munir , S. Habibzadeh , A. Salmankhani , F. J. Stadler , M. R. Saeb , J. Compos. Sci. 2020, 4, 75.

[2] J. Xia , Y. X. Gao , G. Yu , J. Colloid Interface Sci. 2021, 590, 495. 33567374 10.1016/j.jcis.2021.01.046

[3] X. Zhao , Y. Wang , D.‐S. Li , X. Bu , P. Feng , Adv. Mater. 2018, 30, 1705189. 10.1002/adma.201705189 29582482

[4] R. B. Lin , S. C. Xiang , W. Zhou , B. L. Chen , Chem 2020, 6, 337.

[5] Y. J. Qin , X. Han , Y. P. Li , A. J. Han , W. X. Liu , H. J. Xu , J. F. Liu , ACS Catal. 2020, 10, 5973.

[6] W. Y. Gao , A. D. Cardenal , C. H. Wang , D. C. Powers , Chem. ‐ Eur. J. 2019, 25, 3465. 30335210 10.1002/chem.201804490

[7] Y. Yan , Z. Shi , H. Li , L. Li , X. Yang , S. Li , H. Liang , Z. Qiao , Chem. Eng. J. 2022, 427, 131604.

[8] L. Li , T. Zhang , Y. Duan , Y. Wei , C. Dong , L. Ding , Z. Qiao , H. Wang , J. Mater. Chem. A 2018, 6, 11734.

[9] K. H. Li , D. H. Olson , J. Seidel , T. J. Emge , H. W. Gong , H. P. Zeng , J. Li , J. Am. Chem. Soc. 2009, 131, 10368. 19722614 10.1021/ja9039983

[10] Y. Liu , Z. Chen , G. Liu , Y. Belmabkhout , K. Adil , M. Eddaoudi , W. Koros , Adv. Mater. 2019, 31, 1807513. 10.1002/adma.201807513 30768815

[11] D. S. Sholl , Accounts Chem. Res. 2006, 39, 403. 10.1021/ar0402199 16784218

[12] A. I. Skoulidas , D. S. Sholl , J. Phys. Chem. B 2005, 109, 15760. 16853000 10.1021/jp051771y

[13] H. Jobic , J. Karger , M. Bee , Phys. Rev. Lett. 1999, 82, 4260.

[14] R. Thoma , J. Karger , N. D. Amadeu , S. Niessing , C. Janiak , Chemistry 2017, 23, 13000. 28722297 10.1002/chem.201702586

[15] C. Chmelik , D. Freude , H. Bux , J. Haase , Microporous Mesoporous Mat. 2012, 147, 135.

[16] S. Brandani , J. Hufton , D. Ruthven , Zeolites 1995, 15, 624.

[17] Y. G. Chung , J. Camp , M. Haranczyk , B. J. Sikora , W. Bury , V. Krungleviciute , T. Yildirim , O. K. Farha , D. S. Sholl , R. Q. Snurr , Chem. Anal. Biol. Fate: Polynucl. Aromat. Hydrocarbons 2014, 26, 6185.

[18] Y. G. Chung , E. Haldoupis , B. J. Bucior , M. Haranczyk , S. Lee , H. Zhang , K. D. Vogiatzis , M. Milisavljevic , S. Ling , J. S. Camp , B. Slater , J. I. Siepmann , D. S. Sholl , R. Q. Snurr , J. Chem. Eng. Data 2019, 64, 5985.

[19] C. E. Wilmer , M. Leaf , C. Y. Lee , O. K. Farha , B. G. Hauser , J. T. Hupp , R. Q. Snurr , Nat. Chem. 2012, 4, 83. 10.1038/nchem.1192 22270624

[20] T. Chokbunpiam , R. Chanajaree , J. Caro , W. Janke , T. Remsungnen , S. Hannongbua , S. Fritzsche , Comput. Mater. Sci. 2019, 168, 246.

[21] I. B. Orhan , H. Daglar , S. Keskin , T. C. Le , R. Babarao , ACS Appl. Mater. Interfaces 2022, 14, 736. 34928569 10.1021/acsami.1c18521

[22] Y. Wu , H. Duan , H. Xi , Chem. Mater. 2020, 32, 2986.

[23] S. M. Lundberg , G. Erion , H. Chen , A. DeGrave , J. M. Prutkin , B. Nair , R. Katz , J. Himmelfarb , N. Bansal , S. I. Lee , Nat Mach Intell 2020, 2, 56. 32607472 10.1038/s42256-019-0138-9 PMC7326367

[24] S. M. Lundberg , S. I. Lee , A Unified Approach to Interpreting Model Predictions, Neural Information Processing Systems (Nips), Long Beach, CA 2017.

[25] Z. Wang , T. Zhou , K. Sundmacher , Chem. Eng. J. 2022, 444, 136651.

[26] S. M. Lundberg , B. Nair , M. S. Vavilala , M. Horibe , M. J. Eisses , T. Adams , D. E. Liston , D. K. W. Low , S. F. Newman , J. Kim , S. I. Lee , Nat Biomed Eng 2018, 2, 749. 31001455 10.1038/s41551-018-0304-0 PMC6467492

[27] M. I. M. Kusdhany , S. M. Lyth , Carbon 2021, 179, 190.

[28] T. Onsree , N. Tippayawong , S. Phithakkitnukoon , J. Lauterbach , Energy 2022, 249, 11.

[29] A. K. Rappi , C. J. Casewit , K. S. Colwell , W. A. G. III , W. M. Skid , J. Am. Chem. Soc. 1992, 114, 10024.

[30] E. S. Kadantsev , P. G. Boyd , T. D. Daff , T. K. Woo , J. Phys. Chem. Lett. 2013, 4, 3056.

[31] H. Daglar , S. Keskin , J. Phys. Chem. C 2018, 122, 17347. 10.1021/acs.jpcc.8b05416 PMC6077770 30093931

[32] T. F. Willems , C. H. Rycroft , M. Kazi , J. C. Meza , M. Haranczyk , Microporous Mesoporous Mater. 2012, 149, 134.

[33] D. Dubbeldam , S. Calero , D. E. Ellis , R. Q. Snurr , Mol. Simul. 2015, 42, 81.

[34] J. J. Potoff , J. I. Siepmann , AIChE J. 2001, 47, 1676.

[35] M. S. Shah , M. Tsapatsis , J. I. Siepmann , J. Phys. Chem. B 2015, 119, 7041. 25981731 10.1021/acs.jpcb.5b02536

[36] C. Altintas , G. Avci , H. Daglar , E. Gulcay , I. Erucar , S. Keskin , J. Mater. Chem. A 2018, 6, 5836. 10.1039/c8ta01547c PMC6003548 30009024

[37] K. Mukherjee , Y. J. Colon , Mol. Simul. 2021, 47, 857.

[38] H. Daglar , S. Keskin , ACS Appl. Mater. Interfaces 2022, 14, 32134. 35818710 10.1021/acsami.2c08977 PMC9305976

[39] G. Avci , I. Erucar , S. Keskin , ACS Appl. Mater. Interfaces 2020, 12, 41567. 32818375 10.1021/acsami.0c12330 PMC7591111

[40] H. B. Huang , H. Sato , T. Aida , J. Am. Chem. Soc. 2017, 139, 8784. 28635263 10.1021/jacs.7b02979

[41] J. R. Li , R. J. Kuppler , H. C. Zhou , Chem. Soc. Rev. 2009, 38, 1477. 19384449 10.1039/b802426j

[42] F. F. Yin , X. Y. Shao , L. J. Zhao , X. P. Li , J. Y. Zhou , Y. Cheng , X. J. He , S. Lei , J. G. Li , J. L. Wang , Oncol Lett 2019, 18, 1597. 31423227 10.3892/ol.2019.10504 PMC6607378

[43] C. Qi , A. Fourie , X. Zhao , J. Comput. Civ. Eng. 2018, 32, 04018031.

[44] T. Q. Chen , C. Guestrin , M. Assoc Comp , In XGBoost: A Scalable Tree Boosting System, Assoc Computing Machinery, San Francisco, CA 2016, 785.

[45] G. L. Ke , Q. Meng , T. Finley , T. F. Wang , W. Chen , W. D. Ma , Q. W. Ye , T. Y. Liu , In LightGBM: A Highly Efficient Gradient Boosting Decision Tree, Neural Information Processing Systems (Nips), Long Beach, CA 2017.

[46] G. C. Cawley , N. L. C. Talbot , J. Mach. Learn. Res. 2010, 11, 2079.

[47] D. S. Soper , Electronics 2021, 10, 23.

[48] Y. Jung , J. Nonparametr. Stat. 2018, 30, 197.

[49] R. Jaafreh , Y. S. Kang , K. Hamad , ACS Appl. Mater. Interfaces 2021, 13, 57204. 34806862 10.1021/acsami.1c17378

[50] S. Shekhar , A. Bansode , A. Salim , A Comparative study of Hyper‐Parameter Optimization Tools, 2021 IEEE Asia‐Pacific Conference on Computer Science and Data Engineering (CSDE) , 2021.

[51] K. Fujimoto , I. Kojadinovic , J.‐L. Marichal , Games Econ. Behav. 2006, 55, 72.

[52] X. N. Bai , Z. A. Shi , H. Xia , S. H. Li , Z. L. Liu , H. Liang , Z. T. Liu , B. F. Wang , Z. W. Qiao , Chem. Eng. J. 2022, 446, 136783.

[53] S. Lipovetsky , M. Conklin , Appl. Stoch. Models. Bus. Ind. 2001, 17, 319.

[54] Z. A. Shi , W. Y. Yang , X. M. Deng , C. Z. Cai , Y. L. Yan , H. Liang , Z. L. Liu , Z. W. Qiao , Mol. Syst. Des. Eng. 2020, 5, 725.

[55] J. Yan , Y. T. Xu , Q. Cheng , S. Q. Jiang , Q. Wang , Y. J. Xiao , C. Ma , J. B. Yan , X. F. Wang , Genome. Biol. 2021, 22, 24.33461601

[56] W. Z. Liang , S. Z. Luo , G. Y. Zhao , H. Wu , Mathematics 2020, 8, 17.

[57] Z. W. Qiao , C. W. Peng , J. Zhou , J. W. Jiang , J. Mater. Chem. A 2016, 4, 15904.

[58] T. H. Hung , Q. Lyu , L. C. Lin , D. Y. Kang , J. Phys. Chem. C 2021, 125, 20416.

[59] M. S. Zhou , A. Vassallo , J. Z. Wu , J. Membr. Sci. 2020, 598, 117675.

[60] S. Budhathoki , O. Ajayi , J. A. Steckel , C. E. Wilmer , Energy Environ. Sci. 2019, 12, 1255.

[61] S. Yu , J. Bo , M. J. Qu , Energy Fuels 2018, 32, 3085.

[62] J. M. Lucero , M. A. Carreon , ACS Appl. Mater. Interfaces 2020, 12, 32182. 32568506 10.1021/acsami.0c08040

[63] C. H. Jiang , X. K. Wang , Y. G. Ouyang , K. B. Lu , W. F. Jiang , H. K. Xu , X. F. Wei , Z. F. Wang , F. N. Dai , D. F. Sun , Nanoscale Adv. 2022, 4, 2077. 36133454 10.1039/d2na00061j PMC9418345

[64] R. Krishna , RSC Adv. 2015, 5, 52269.

[65] C. Altintas , S. Keskin , Mol. Syst. Des. Eng. 2020, 5, 532.

[66] T. H. Hung , X. P. Deng , Q. Lyu , L. C. Lin , D. Y. Kang , J. Membr. Sci. 2021, 639, 119742.

[67] T. M. Becker , J. Heinen , D. Dubbeldam , L. C. Lin , T. J. H. Vugt , J. Phys. Chem. C 2017, 121, 4659. 10.1021/acs.jpcc.6b12052 PMC5338003 28286598

[68] G. Borboudakis , T. Stergiannakos , M. Frysali , E. Klontzas , I. Tsamardinos , G. E. Froudakis , npj Comput. Mater. 2017, 3, 7.

[69] J. Liu , D. M. Strachan , P. K. Thallapally , Chem. Commun. 2014, 50, 466. 10.1039/c3cc47777k 24256738

[70] K. Jiang , L. Zhang , T. F. Xia , Y. Yang , B. Li , Y. J. Cui , G. D. Qian , Sci. China‐Mater. 2019, 62, 1315.

[71] T.‐H. Kim , S.‐Y. Kim , T.‐U. Yoon , M.‐B. Kim , W. Park , H. H. Han , C.‐i. Kong , C.‐Y. Park , J.‐H. Kim , Y.‐S. Bae , Chem. Eng. J. 2020, 399, 125717.

[72] A. Modak , A. Ghosh , A. Bhaumik , B. Chowdhury , Adv. Colloid Interface Sci. 2021, 290, 102349. 33780826 10.1016/j.cis.2020.102349

[73] T. T. Zhao , Y. J. Hui , Niamatullah , Z. H. Li , Mol. Catal. 2019, 474, 110421.

[74] Q. Wang , D. Astruc , Chem. Rev. 2020, 120, 1438. 31246430 10.1021/acs.chemrev.9b00223

[75] H. L. Lu , X. Z. Yang , G. J. Gao , J. Wang , C. H. Han , X. Y. Liang , C. F. Li , Y. Y. Li , W. D. Zhang , X. T. Chen , Fuel 2016, 183, 335.

[76] M. Mihet , O. Grad , G. Blanita , T. Radu , M. D. Lazar , Int. J. Hydrog. Energy 2019, 44, 13383.

[77] W. L. Zhen , B. Li , G. X. Lu , J. T. Ma , Chem. Commun. 2015, 51, 1728. 10.1039/c4cc08733j 25518948

[78] W. K. Fan , M. Tahir , Ind. Eng. Chem. Res. 2021, 60, 13149.

