# Supplementary material for: Interpretable Machine‐Learning and Big Data Mining to Predict Gas Diffusivity in Metal‐Organic Frameworks
Source: Adv Sci (Weinh). 2023 May 11;10(21):2301461. doi: 10.1002/advs.202301461 (PMC10375163; doi:10.1002/advs.202301461)
Supplement: Supplementary file 1 — Supporting Information [file ADVS-10-2301461-s001.pdf]

ADVANCED SCIENCE
Open Access

Supporting Information

for *Adv. Sci.*, DOI 10.1002/advs.202301461

Interpretable Machine-Learning and Big Data Mining to Predict Gas Diffusivity in Metal-Organic Frameworks

Shuya Guo, Xiaoshan Huang, Yizhen Situ, Qiuhong Huang, Kexin Guan, Jiaxin Huang, Wei Wang, Xiangning Bai, Zili Liu, Yufang Wu and Zhiwei Qiao**

Supporting Information

Interpretable Machine-Learning and Big Data Mining to Predict Gas Diffusivity in Metal-Organic Frameworks

Shuya Guo, Xiaoshan Huang, Yizhen Situ, Qiuhong Huang, Kexin Guan, Jiaxin Huang, Wei Wang, Xiangning Bai, Zili Liu, Yufang Wu, and Zhiwei Qiao**

Table of contents:

Section

S1 Adsorbate Force Field Parameters ……………………………………. S2
S2 Characteristics of gas molecules ……………………………………… S4
S3 Details of Model Training …………………………………………….. S6
S4 Evaluation of machine learning ………………………………………. S14
S5 Analysis of the relative importance of features ………………………. S18
S6 Top-performing MOFs ………………………………………………... S23
S7 Additional Extrapolated Adsorbate Prediction Data ………………….. S25
References …………………………………………………………………... S32

Section S1. Adsorbate Force Field Parameters

Table S1. Lennard–Jones parameters of MOF[1]

Atom	ε/k_B [K]	σ [Å]	Atom	ε/k_B [K]	σ [Å]	Atom	ε/k_B [K]	σ [Å]
Ac	16. 60	3. 10	Ge	190. 69	3. 81	Po	163. 52	4. 20
Ag	18. 11	2. 80	Gd	4. 53	3. 00	Pr	5. 03	3. 21
Al	254. 09	4. 01	H	22. 14	2. 57	Pt	40. 25	2. 45
Am	7. 04	3. 01	Hf	36. 23	2. 80	Pu	8. 05	3. 05
Ar	93. 08	3. 45	Hg	193. 71	2. 41	Ra	203. 27	3. 28
As	155. 47	3. 77	Ho	3. 52	3. 04	Rb	20. 13	3. 67
At	142. 89	4. 23	I	170. 57	4. 01	Re	33. 21	2. 63
Au	19. 62	2. 93	In	301. 39	3. 98	Rh	26. 67	2. 61
B	90. 57	3. 64	Ir	36. 73	2. 53	Rn	124. 78	4. 25
Ba	183. 15	3. 30	K	17. 61	3. 40	Ru	28. 18	2. 64
Be	42. 77	2. 45	Kr	110. 69	3. 69	S	137. 86	3. 59
Bi	260. 63	3. 89	La	8. 55	3. 14	Sb	225. 91	3. 94
Bk	6. 54	2. 97	Li	12. 58	2. 18	Sc	9. 56	2. 94
Br	126. 29	3. 73	Lu	20. 63	3. 24	Se	146. 42	3. 75
C	52. 83	3. 43	Lr	5. 53	2. 88	Si	202. 27	3. 83
Ca	119. 75	3. 03	Md	5. 53	2. 92	Sm	4. 03	3. 14
Cd	114. 72	2. 54	Mg	55. 85	2. 69	Sn	285. 28	3. 91
Ce	6. 54	3. 17	Mn	6. 54	2. 64	Sr	118. 24	3. 24
Cf	6. 54	2. 95	Mo	28. 18	2. 72	Ta	40. 75	2. 82
Cl	114. 21	3. 52	N	34. 72	3. 26	Tb	3. 52	3. 07
Cm	6. 54	2. 96	Na	15. 09	2. 66	Tc	24. 15	2. 67
Co	7. 04	2. 56	Ne	21. 13	2. 66	Te	200. 25	3. 98
Cr	7. 55	2. 69	Nb	29. 69	2. 82	Th	13. 08	3. 03
Cu	2. 52	3. 11	Nd	5. 03	3. 18	Ti	8. 55	2. 83
Cs	22. 64	4. 02	No	5. 53	2. 89	TI	342. 14	3. 87
Dy	3. 52	3. 05	Ni	7. 55	2. 52	Tm	3. 02	3. 01
Eu	4. 03	3. 11	Np	9. 56	3. 05	U	11. 07	3. 02
Er	3. 52	3. 02	O	30. 19	3. 12	V	8. 05	2. 80
Es	6. 04	2. 94	Os	18. 62	2. 78	W	33. 71	2. 73
F	25. 16	3. 00	P	153. 46	3. 69	Xe	167. 04	3. 92
Fe	6. 54	2. 59	Pa	11. 07	3. 05	Y	36. 23	2. 98
Fm	6. 04	2. 93	Pb	333. 59	3. 83	Yb	114. 72	2. 99
Fr	25. 16	4. 37	Pd	24. 15	2. 58	Zn	62. 39	2. 46
Ga	208. 81	3. 90	Pm	4. 53	3. 16	Zr	34. 72	2. 78

Table S2. Lennard–Jones parameters and charges of adsorbates[2, 3]

Atom	ε/k_B [K]	σ [Å]	Charge (e)	Atom	ε/k_B [K]	σ [Å]	Charge (e)
C_CO_2	27.0	2.80	+0.700	S_H_2S	122.0	3.60	0
O_CO_2	79.0	3.05	–0.350	M_H_2S	0	0	–0.420
CH_4	148.0	3.73	0	H_H_2	0	0	+0.468
N_N_2	36.0	3.31	–0.482	com_H_2	36.7	2.96	–0.936
com_N_2	0	0	+0.964	He- He	10.9	2.64	0
O_O_2	49.0	3.02	–0.113	Kr	166.4	3.636	0
com _O_2	0	0	+0.226	Xe	221.0	4.10	0
H_H_2S	50.0	2.50	+0.210	CH3	98.0	3.750	0

The molecular diffusion coefficient for the calculation of MD simulation method has been used and compared with experiments in a series of previous studies,[4-14] which has been demonstrated as accurate. The simulated and experimental diffusion coefficients and permeabilities are similar in Figure S1.

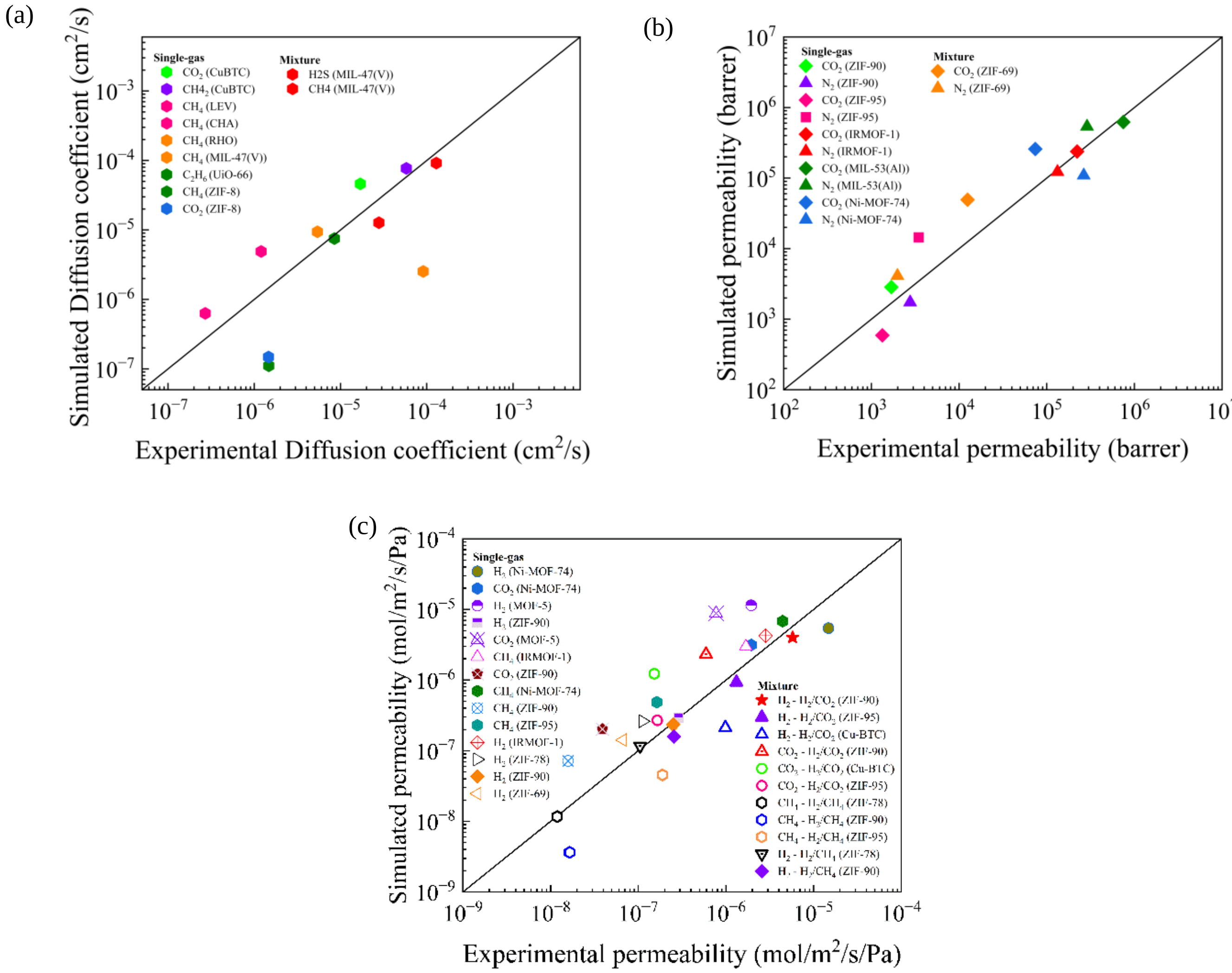

Figure S1. (a) Comparison of simulated gas diffusivities and the experimental data for various MOF.[5-8, 15] (b) and (c) Comparison of simulated gas permeabilities and the experimental data for various MOF membrabes.[9, 10]

Section S2. Characteristics of gas molecules

Table S3. Physical properties of gas molecules.[16](* stands for predictive molecule)

Gas	Kinetic diameter [Å]	Polarizability [×10^{25} /cm^3]	Dipole moment [×10^{18} /esu cm]	Quadruple moment [×10^{26} /esu cm^2]
He	2.6	2.04956	0	0
H_2	2.89	8.042	0	0.662
CO_2	3.3	29.11	0	4.30
O_2	3.46	15.812	0	0.39
H_2S	3.62	37.82	0.97833	—
N_2	3.64	17.403	0	1.52
kr	3.66	24.844	0	0
CH_4	3.758	25.93	0	0
Xe	4.1	40.44	0	0
C_2H_{6*}	4.443	44.5	0	0.65

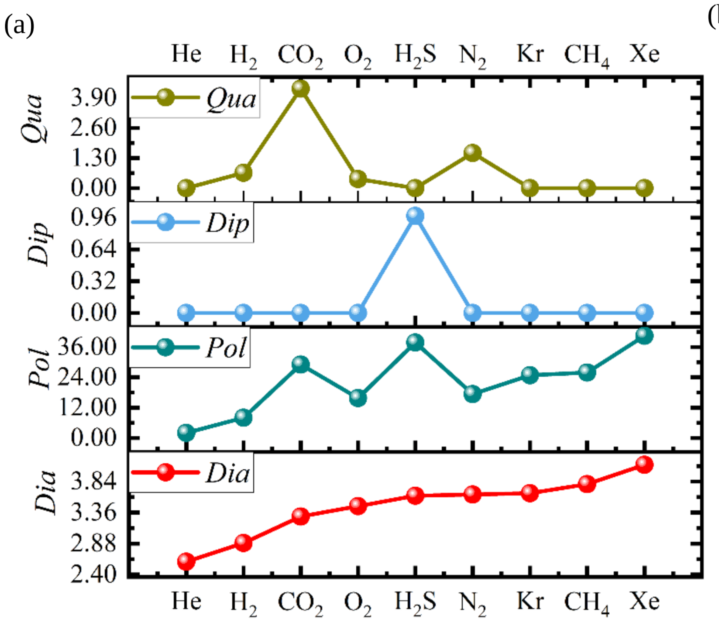

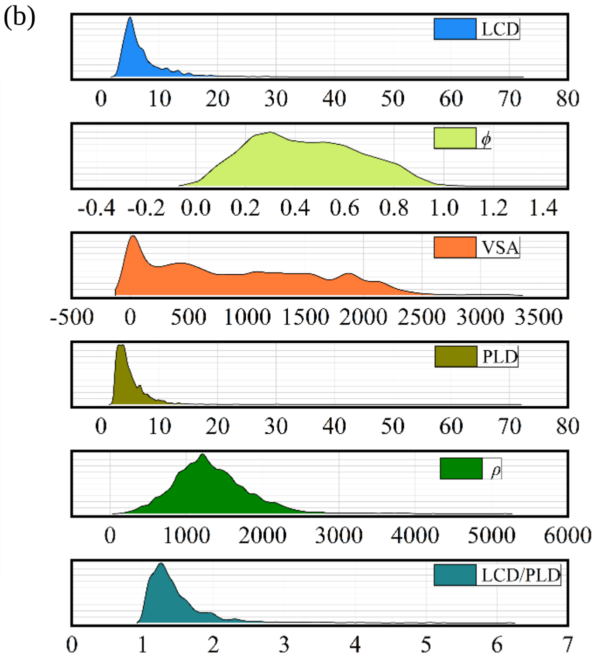

Figure S2. (a) Physical properties of gas molecules. (b) Distribution of physical descriptors for the 2019 CoRE-MOFs database.

Table S4. Differences of kinetic diameter, polarizability, dipole moment, and quadruple moment between binary gas mixtures.

	Gas mixture *i/j*	Δ*Dia* [Å]	Δ*Pol* [×10^{25}/cm^3]	Δ*Dip* [×10^{18}/esu cm]	Δ*Qua* [×10^{26}/esucm^2]
In Molecular Simulation	He/H_2	0.29	5.99244	0	0.662
	He/CO_2	0.7	27.06044	0	4.3
	He/O_2	0.86	13.76244	0.97833	0.39
	He/H_2S	1.02	36.61044	0.97833	0
	He/N_2	1.04	15.35344	0	1.52
	He/Kr	1.06	22.79444	0	0
	He/CH_4	1.2	23.88044	0	0
	He/Xe	1.5	38.39044	0	0
	H_2/CO_2	0.41	21.068	0	3.638
	H_2/O_2	0.57	7.77	0	-0.272
	H_2/H_2S	0.73	30.618	0.97833	-0.662
	H_2/N_2	0.75	9.361	0	0.858
	H_2/kr	0.77	16.802	0	-0.662
	H_2/CH_4	0.91	17.888	0	-0.662
	H_2/Xe	1.21	32.398	0	-0.662
	CO_2/O_2	0.16	-13.298	0	-3.91
	CO_2/H_2S	0.32	9.55	0.97833	-4.3
	CO_2/N_2	0.34	-11.707	0	-2.78
	CO_2/kr	0.36	-4.266	0	-4.3
	CO_2/CH_4	0.5	-3.18	0	-4.3
	CO_2/Xe	0.8	11.33	0	-4.3
	O_2/H_2S	0.16	22.848	0.97833	-0.39
	O_2/N_2	0.18	1.591	0	1.13
	O_2/kr	0.2	9.032	0	-0.39
	O_2/CH4	0.34	10.118	0	-0.39
	O_2/Xe	0.64	24.628	0	-0.39
	H_2S/N_2	0.02	-21.257	-0.97833	1.52
	H_2S/kr	0.04	-13.816	-0.97833	0
	H_2S/CH_4	0.18	-12.73	-0.97833	0
	H_2S/Xe	0.48	1.78	-0.97833	0
	N_2/kr	0.02	7.441	0	-1.52
	N_2/CH_4	0.16	8.527	0	-1.52
	N_2/Xe	0.46	23.037	0	-1.52
	Kr/CH_4	0.14	1.086	0	0
	Kr/Xe	0.44	15.596	0	0
	CH_4/Xe	0.3	14.51	0	0
ML Prediction	He/C_2H_6	1.843	42.45044	0	0.65
	H_2/C_2H6	1.553	36.458	0	-0.012
	CO_2/C_2H_6	1.143	15.39	0	-3.65
	O_2/C_2H6	0.983	28.688	0	0.26
	C_2H_6/H_2S	-0.823	-6.68	0.97833	-0.65
	N_2/C_2H_6	0.803	27.097	0	-0.87
	C_2H_6/Kr	-0.783	-19.656	0	-0.65

C_2H_6/CH_4	-0.643	-18.57	0	-0.65

Pearson correlation coefficient:

The Pearson product-moment correlation coefficient (PPMCC or PCCs), which varies in value from -1 to 1, is used to calculate the correlation between the two variables, X and Y. A Pearson product-moment correlation coefficient between two variables is defined as the quotient of covariance and standard deviation between two variables:

$$r=\frac{\sum_{i=1}^{n}(x_i-\bar{x})(Y_i-\bar{Y})}{\sqrt{\sum_{i=1}^{n}(X_i-\bar{x})^2}\sqrt{\sum_{i=1}^{n}(Y_i-\bar{Y})^2}}$$

Section S3. Details of Model Training

Python 3.9.12 was used for all training tasks.

Table S5. The version information of tool packages used for building ML model

Package	Version
sklearn	1.02
numpy	1.21.5
random	1.2.2
pandas	1.3.5
shap	0.40.0
lightgbm	3.3.2
xgboost	1.1.2
joblib	1.1.0

Overview of four machine learning algorithms

In this work, we utilize four different ML methods (RF, GBRT, XGBoost, and LightGBM). They are all outcomes of the ensemble learning theory. Ensemble learning addresses inherent flaws in a single model or a model with a certain set of parameters. Its fundamental idea is to combine weak learners to establish a strong model. The Bagging and Boosting methods comprise most of the ensemble learning methods. The full name of Bagging is "bootstrap aggregation". The working mechanism is shown in Figure S3, the basic idea of Bagging is to train multiple classifiers, and there is no strong dependence between the classifiers, then calculate the average of the calculated results. RF algorithm is an evolution of Bagging, based on the random sampling of bagging samples, RF algorithm also adds a random selection of features, and its basic idea is not divorced from the category of bagging. However, The Boosting Method is based on a serial strategy, and the new learner is generated by the old learner. The working mechanism is displayed in Figure S4. The same training is used for all sample weights to build the first weak classifier, followed by an adjustment of the sample weight based on the results of the previous classification. Points near the classification border receive greater weights since they are more likely to be misclassified. These are all typical examples of boosting: GBRT, XGBoost, and LightGBM. The four algorithms' main differences, advantages, and disadvantages are listed below: RF, GBRT, XGBoost, and LightGBM. (A summary of the following algorithms, excluding classification algorithms, is

based on the introduction of the regression models used in this work.)

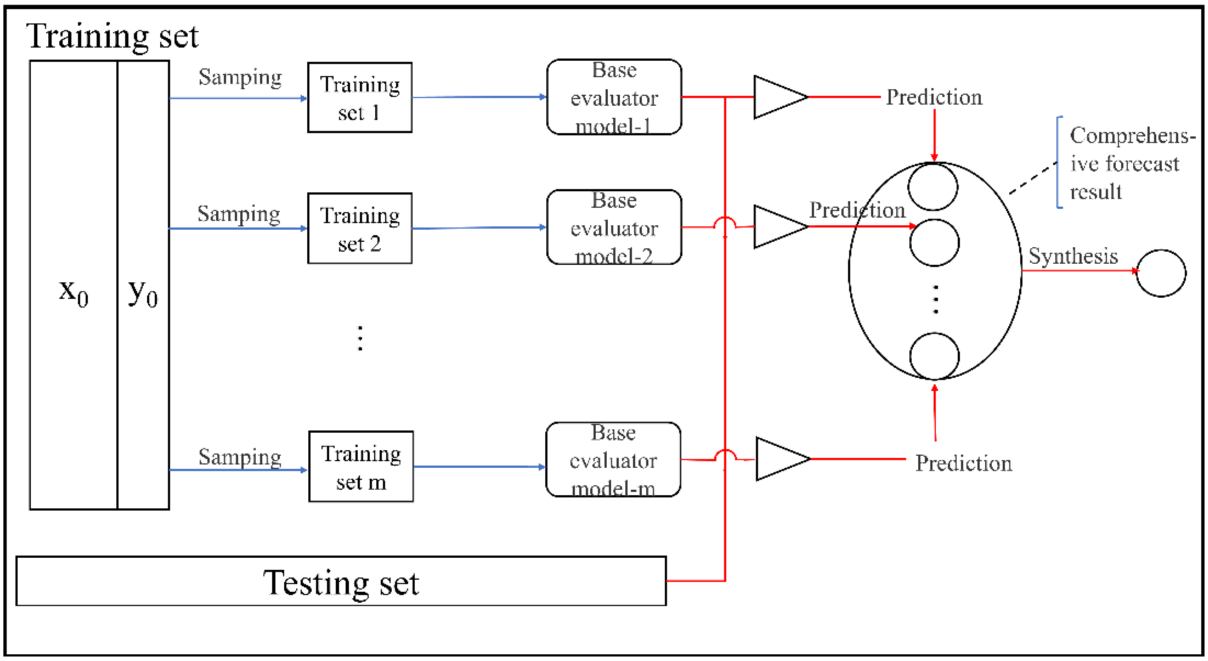

Figure S3. Bagging model.

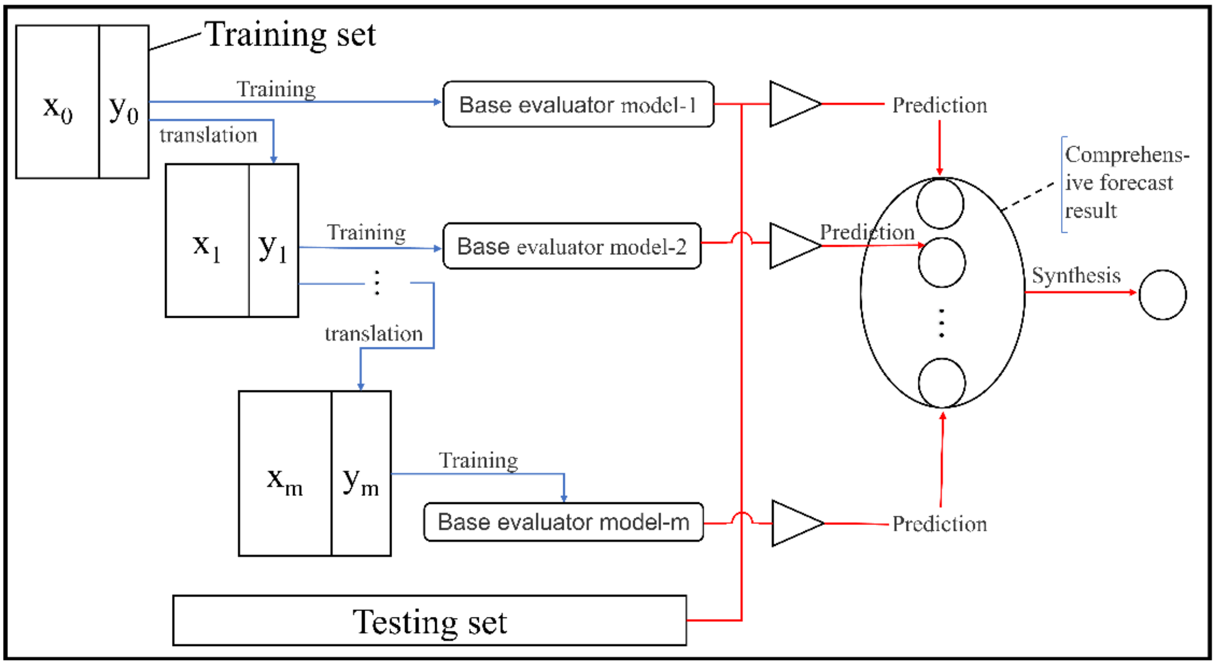

Figure S4. Boosting model.

Random Forest (RF) Algorithm

As an extension of the Bagging variations, built using decision trees as the learner, random forest (RF) further integrates random feature selection into the decision tree training process. It may be boiled down into four sections, as shown in Figure S5: a new training sample set is made by taking repeated random samples of k from the initial training sample set of N. In this study, the feature characteristics are chosen at random, and all the features are trained; the predictions of a single decision tree are obtained based on the sample extraction; the predictions from all decision trees are then averaged to provide the final predictions.[17] The random forest algorithm has the benefits of strong generalizability, the capacity to handle missing data, and the ability to be used without normalization. However, it can only generate predictions inside the training set, which causes overfitting when modeling some noisy data.

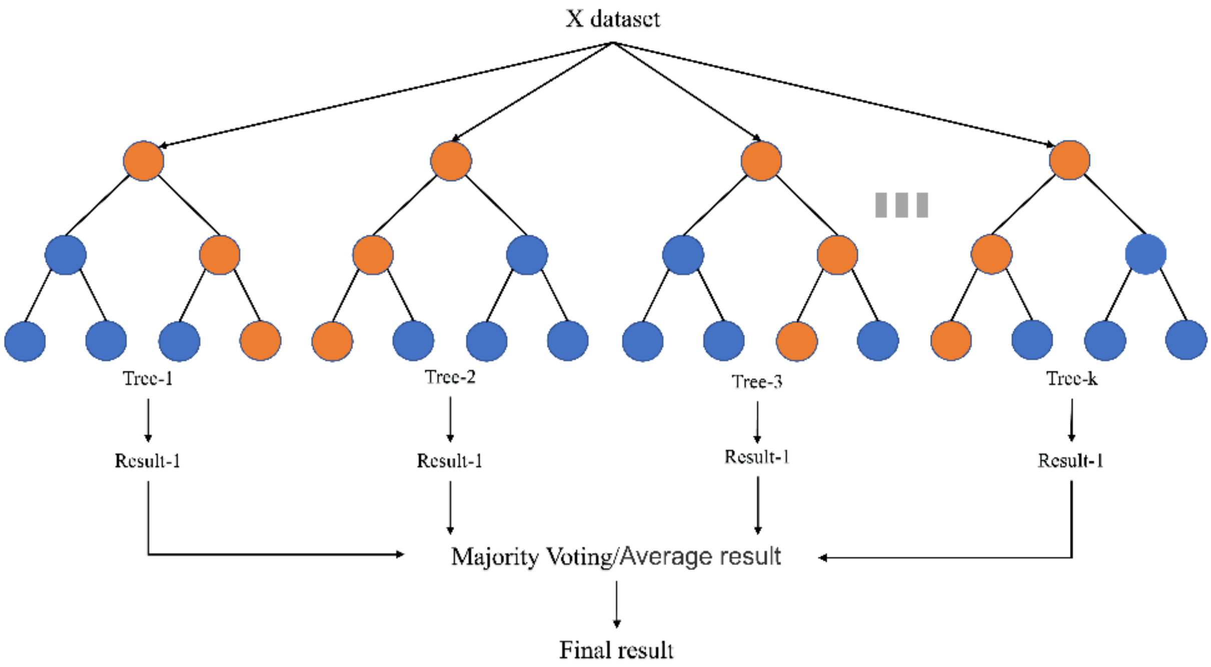

Figure S5. Random Forest model.

Gradient boosting regression tree Algorithm

Gradient boost is the fundamental tenet of the Gradient boosting regression tree (GBRT). Gradient Boosting is a strategy for boosting, and it differs from traditional boosting in that each calculation is performed to lower the residual of the previous one. And to eliminate the residual, a new model will be constructed in the direction of the Gradient in which the residual is reduced. In support of the forecast derivation, the final iteration of the gradient learning loss function is used. As illustrated in Figure S6, the "squared error" loss function used in this study produces a continuous error fit during the learning process. Each regression tree is used to fit the current GBRT by learning the conclusions and residuals of earlier trees. The advantages of GBRT are evident. It can deal with all types of data flexibly, and the prediction accuracy is increased under the relatively short time of parameter change. The performance of GBRT is further enhanced by RF. It is challenging to train data in parallel since the basic learner already has serial relations due to the Boosting architecture.

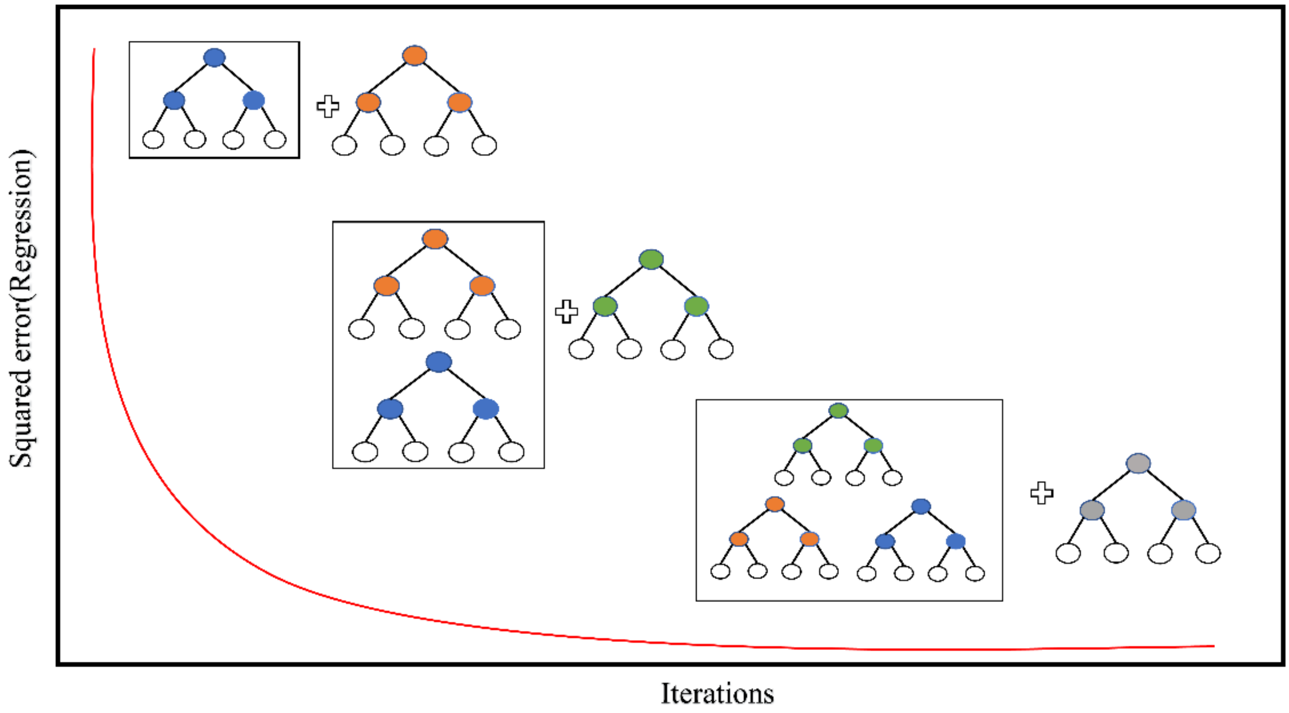

Figure S6. GBRT model.

Extreme Gradient Boosting Algorithm

Extreme Gradient Boosting, or XGBoost, was built by Tianqi Chen of the University of Washington to improve the GBDT-based boosting algorithm. It approximates the residuals using the negative gradient of the model on the data, but it is a Taylor series approximation of the model's loss residuals with the addition of a regular term of model complexity. XGBoost performs better than the GBDT because it utilizes parallel CPUs to run more quicker. The next generation of XGBoost is likewise one iteration away (The price of the first t iteration function contains t-1 in front of the iteration of the predicted values). Use these later rounds of XGBoost frequently to sort the data in preparation and store it in the block structure before training. Parallelism is also feasible by using this block layout. We must first determine the gain of each feature before selecting the one with the highest gain to split nodes. The gain of each feature may then be determined by many threads. However, as shown in Figure S7, the level-wise method is the same for all leaf nodes in the current layer. Even if some of the leaf nodes split very little profit and do not have an impact on the outcome, they nonetheless split, raising the cost of computation.[18]

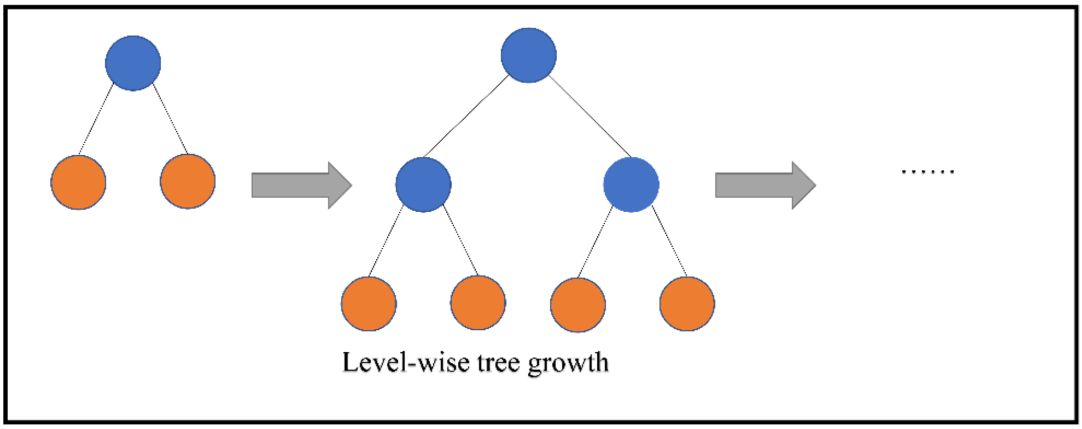

Figure S7. XGBoost model.

LightGBM Algorithm

Another algorithm built by Microsoft Research Asia utilizing the GBDT framework is named LightGBM.[19] It seeks to increase computing effectiveness to address big data prediction challenges more successfully. Lightgbm uses the GOSS (Gradient-based One-Side Sampling) and EFB (Exclusive Feature Bundling) methods for random sampling and feature extraction. EFB does not scan all features to find the best cut-off point; instead, it reduces the dimensionality of features by grouping them, which lowers the cost of locating the best cut-off point.[19] GOSS does not use the sample points used to calculate gradients; instead, samples are sampled to calculate gradients. While accuracy is not compromised and may be improved in certain cases, processing samples takes much less time.

LightGBM utilizes a Histogram algorithm to combine mutually exclusive features. The fundamental principle of the histogram technique is to discretize the continuous eigenvalues into k integers and build a histogram with width k, as shown in Figure S8(b). The Histogram accumulates the statistics based on the discretized values as an index in the Histogram as it traverses the data. The Histogram first traverses the data once to get the appropriate statistics, and then, using the discrete values of the Histogram, the Histogram traverses to choose the best segmentation point. It is possible to lower the cost of calculation and storage using the histogram algorithm. The pre-sorted algorithm is the default in XGBoost, and it requires O(#data) computations, but the Histogram algorithm only needs O(#bins) computations, which is a considerably lesser number than O(#data).

Figure S8(a) shows that LightGBM uses the leaf-wise strategy, whereas XGBoost uses the level-wise split strategy. Find the leaf with the biggest split gain from all the current leaves, split that leaf, and so on. Therefore, with the same number of splits, Leaf-wise can achieve greater accuracy while reducing more mistakes than Level-wise. However, leaf-wise may result in over-fitting when the sample size is small. Therefore, LightGBM may utilize the additional option Max to restrict the depth of the tree and prevent overfitting.[18]

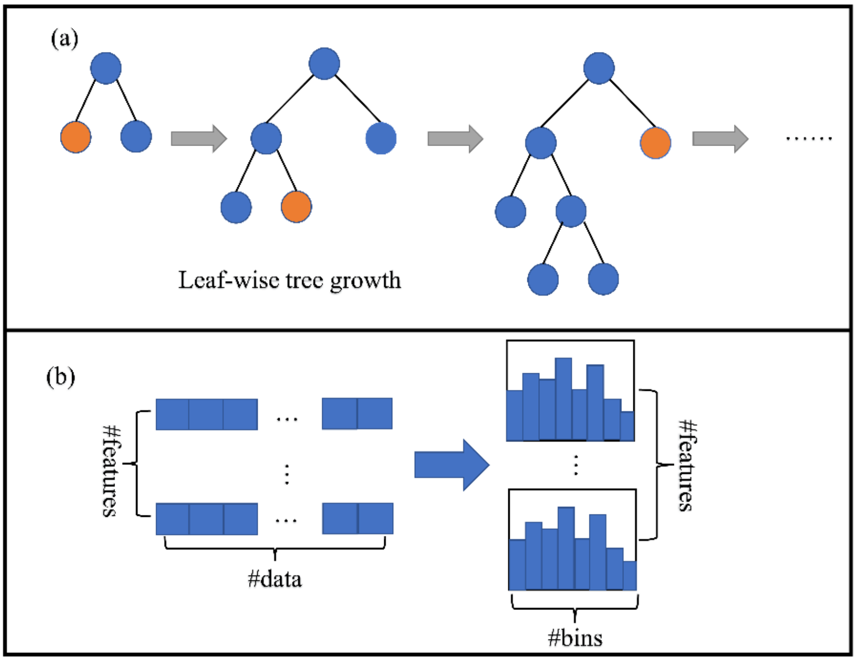

Figure S8. LightGBM model.

Table S6. Hyperparameters set in machine learning methods.

Model	Hyperparameter	Value (On *D*)	Value (On S_{diff})
RF	n_estimators	250	250
	max_depth	14	14
	random_state	90	90
	criterion	'squared_error'	'squared_error'
GBRT	learning_rate	0.1	0.1
	loss	'squared_error'	'squared_error'
	n_estimators	400	500
	subsample	1	1
	criterion	'friedman_mse'	'friedman_mse'
	max_depth	10	12
	alpha	0.5	0.5
	verbose	0	0
	max_leaf_nodes	None	None
	warm_start	False	False
XGBoost	n_estimators	520	650
	max_depth	11	14
	min_child_weight	1	1
	subsample=0.8	0.8	0.8
	gamma=0.0	0	0
	colsample_bytree	0.8	0.8
	nthread	None	None
	reg_alpha	0.8	0.8
	reg_lambda	1	1
	seed	1314	1314
	n_jobs	-1	-1
LightGBM	objective	'regression'	'regression'
	n_estimators	620	1500
	learning_rate	0.1	0.05
	num_leaves	400	1050
	force_col_wise	True	True
	colsample_bytree	0.8	0.8
	subsample_for_bin	90000	220000
	random_state	1314	100
	n_jobs	-1	-1
	min_child_samples	20	20
	reg_alpha	0.6	0
	reg_lambda	0.7	0

Algorithm Evaluation Index

In this work, the performance of each ML algorithm was evaluated by calculating the R^2 values and the root-mean-square error (RMSE). The R^2 value was calculated using eq.S1, where n, y_i, u_i, and $\bar{u}$ are the number of MOFs, the simulated diffusion coefficient of gas molecule (diffusion selectivity of ideal binary gas), the predicted diffusion coefficient of gas molecule (diffusion selectivity of ideal binary gas) and average diffusion coefficient of gas molecule (diffusion selectivity of ideal binary gas), respectively. The various error values for each algorithm were calculated using eq. S2.

$$R^2=1-\frac{\sum_{i=1}^{n}(y_i-u_i)^2}{\sum_{i=1}^{n}(y_i-\bar{u}_i)^2} \quad \text{(S1)}$$

$$\text{RMSE}=\sqrt{\frac{\sum_{i=1}^{n}(y_i-u_i)^2}{n}} \quad \text{(S2)}$$

k-fold cross-validation

It is no longer essential to split the validation set when cross-validation is used, and the test set is always used in the model's final assessment. The cross-validation approach used in this study is known as k-fold cross-validation (k-fold CV), and it divides the training set into k minimum subsets. As shown in Figure S9, one of the k "folds" is used in the method, a subset of k-1 is used for model training, and the remaining data is used to validate the model created in the previous phase (like using the test set to determine the model's accuracy). The results of the k-fold cross-validation, which are the average of the outcomes of the previous stages, serve as a representation of the model's performance.[20] In this work, the 10-fold cross-validation is used.

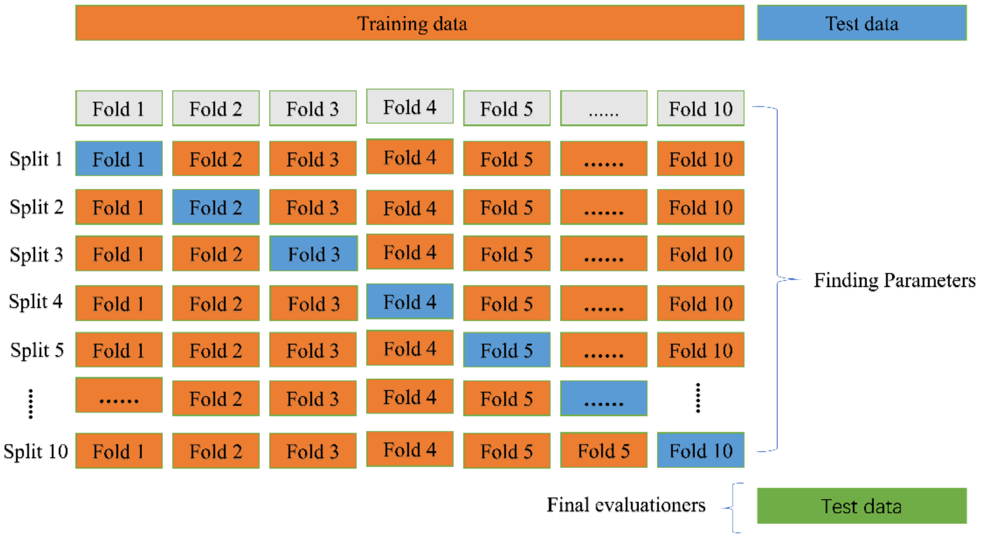

Figure S9. 10-fold cross-validation.

Section S4. Evaluation of machine learning

Table S7. Evaluation of four algorithms for D and S_{diff}.

Algorithm	Performance	CV count	Training set		Cross-validation set	
			R^2	RMSE	R^2	RMSE
RF	D	1	0.970	0.173	0.930	0.261
		2	0.971	0.171	0.933	0.259
		3	0.971	0.172	0.928	0.265
		4	0.971	0.172	0.933	0.258
		5	0.970	0.173	0.939	0.253
		6	0.971	0.171	0.935	0.258
		7	0.970	0.173	0.939	0.247
		8	0.970	0.173	0.935	0.253
		9	0.970	0.173	0.933	0.263
		10	0.971	0.171	0.938	0.251
		Average	0.971	0.172	0.934	0.257
	S_{diff}	1	0.936	0.202	0.889	0.267
		2	0.937	0.202	0.887	0.270
		3	0.936	0.203	0.890	0.266
		4	0.936	0.202	0.889	0.269
		5	0.937	0.201	0.889	0.268
		6	0.936	0.203	0.890	0.264
		7	0.937	0.202	0.886	0.271
		8	0.936	0.203	0.892	0.261
		9	0.936	0.202	0.891	0.265
		10	0.936	0.203	0.893	0.263
		Average	0.936	0.202	0.890	0.266
GBRT	D	1	0.995	0.209	0.955	0.073
		2	0.995	0.202	0.959	0.074
		3	0.995	0.206	0.957	0.074
		4	0.995	0.204	0.958	0.074
		5	0.994	0.195	0.964	0.074
		6	0.995	0.204	0.959	0.074
		7	0.995	0.189	0.964	0.074
		8	0.995	0.196	0.961	0.074
		9	0.995	0.206	0.959	0.072
		10	0.994	0.201	0.961	0.076
		Average	0.995	0.201	0.960	0.074
	S_{diff}	1	0.995	0.056	0.949	0.182
		2	0.995	0.055	0.949	0.181
		3	0.995	0.056	0.952	0.177
		4	0.995	0.057	0.951	0.179
		5	0.995	0.056	0.951	0.178
		6	0.995	0.059	0.949	0.179

	S_{diff}	7	0.995	0.056	0.951	0.177
		8	0.995	0.057	0.951	0.176
		9	0.995	0.055	0.952	0.176
		10	0.995	0.056	0.953	0.175
		Average	0.995	0.056	0.951	0.178
XGBoost	*D*	1	0.993	0.082	0.962	0.192
		2	0.993	0.082	0.964	0.190
		3	0.993	0.081	0.961	0.195
		4	0.993	0.081	0.966	0.184
		5	0.993	0.082	0.968	0.183
		6	0.993	0.082	0.966	0.186
		7	0.993	0.081	0.969	0.176
		8	0.993	0.082	0.967	0.181
		9	0.993	0.082	0.966	0.188
		10	0.993	0.081	0.968	0.182
		Average	0.993	0.082	0.966	0.186
	S_{diff}	1	0.995	0.162	0.959	0.058
		2	0.995	0.162	0.959	0.058
		3	0.995	0.158	0.961	0.058
		4	0.995	0.163	0.959	0.058
		5	0.995	0.160	0.961	0.058
		6	0.995	0.159	0.960	0.058
		7	0.995	0.160	0.960	0.058
		8	0.995	0.156	0.962	0.058
		9	0.995	0.159	0.960	0.058
		10	0.995	0.156	0.962	0.058
		Average	0.995	0.160	0.960	0.058
LGBM	*D*	1	0.994	0.078	0.964	0.188
		2	0.994	0.080	0.966	0.185
		3	0.993	0.082	0.963	0.191
		4	0.993	0.086	0.966	0.183
		5	0.994	0.075	0.968	0.184
		6	0.994	0.078	0.967	0.184
		7	0.993	0.084	0.970	0.174
		8	0.994	0.075	0.968	0.177
		9	0.994	0.078	0.967	0.183
		10	0.993	0.084	0.969	0.179
		Average	0.994	0.080	0.967	0.183
	S_{diff}	1	0.997	0.044	0.970	0.139
		2	0.997	0.044	0.970	0.138
		3	0.997	0.044	0.972	0.135
		4	0.997	0.044	0.971	0.137
		5	0.997	0.044	0.971	0.138
		6	0.997	0.044	0.970	0.137
		7	0.997	0.045	0.971	0.137

	S_{diff}	8	0.997	0.044	0.971	0.135
		9	0.997	0.044	0.971	0.135
		10	0.997	0.044	0.973	0.133
		Average	0.997	0.044	0.971	0.137

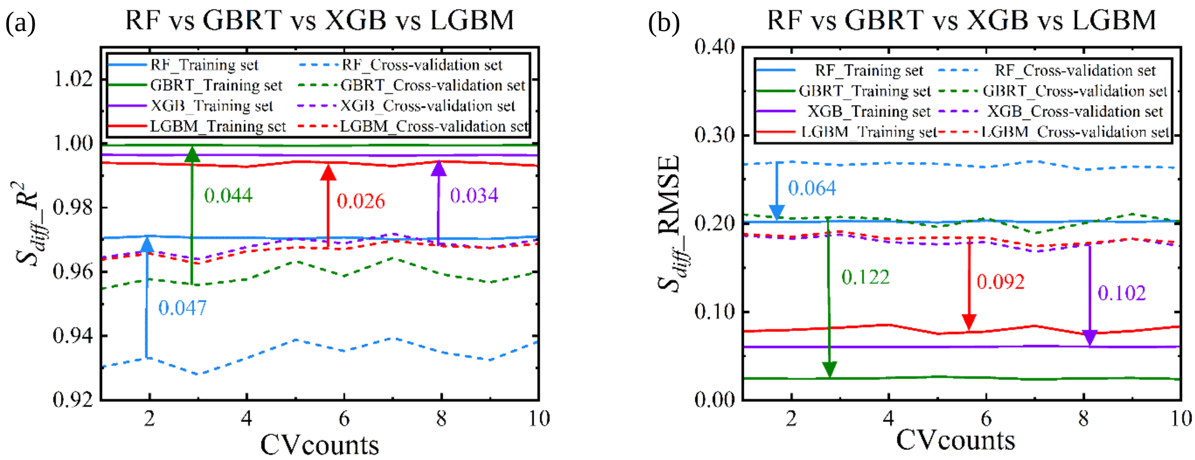

Figure S10. Comparison of performance (R^2, RMSE) of four machine learning algorithms and S_{diff} as the target value under 10-fold cross-validation. (a) and (b) stand for results for R^2 and RMSE, respectively.

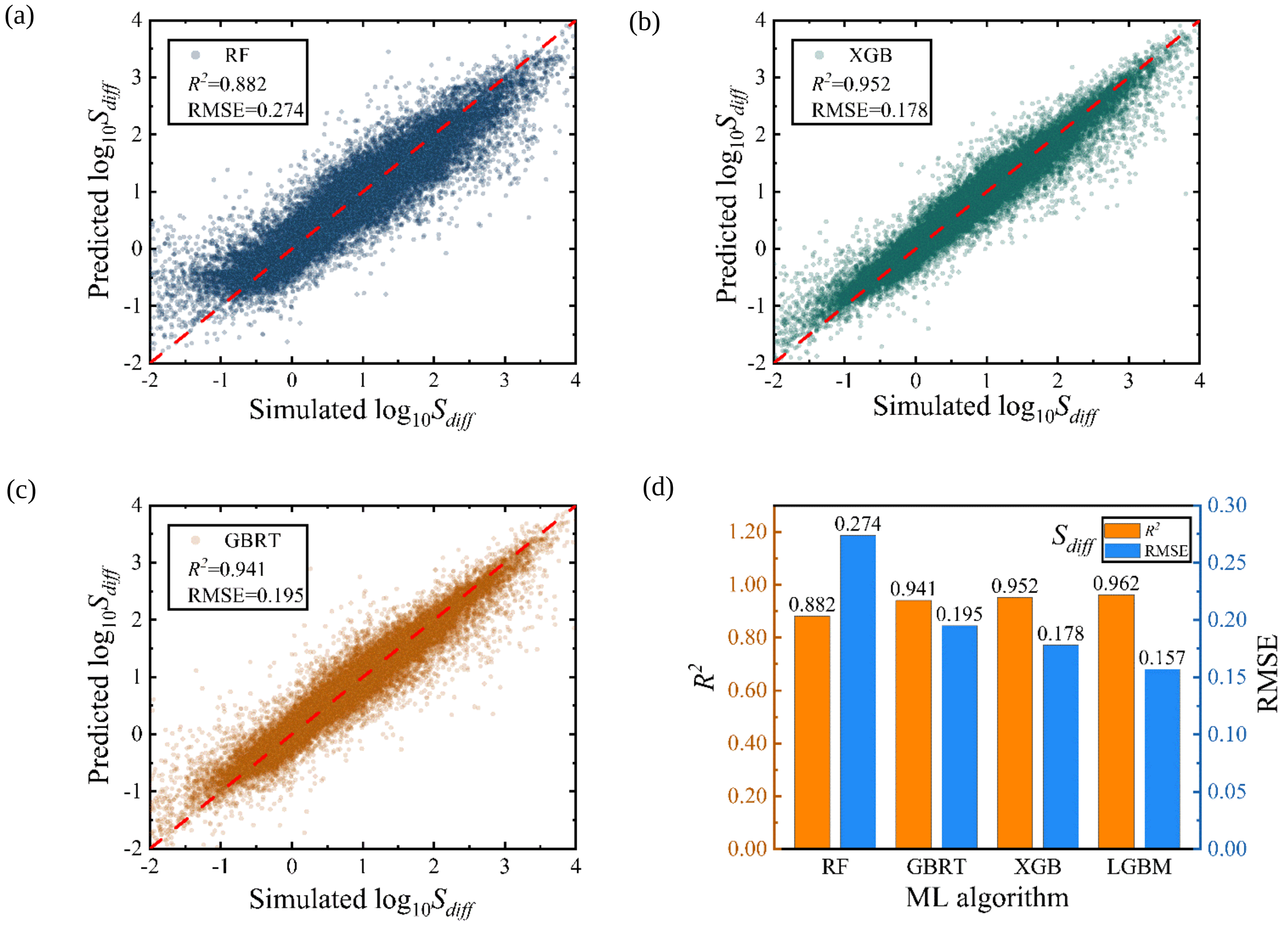

Figure S11. Predicted results of S_{diff} by RF, XGB and GBRT ML algorithm models *versus* simulated results of CoRE-MOFs on the testing set.

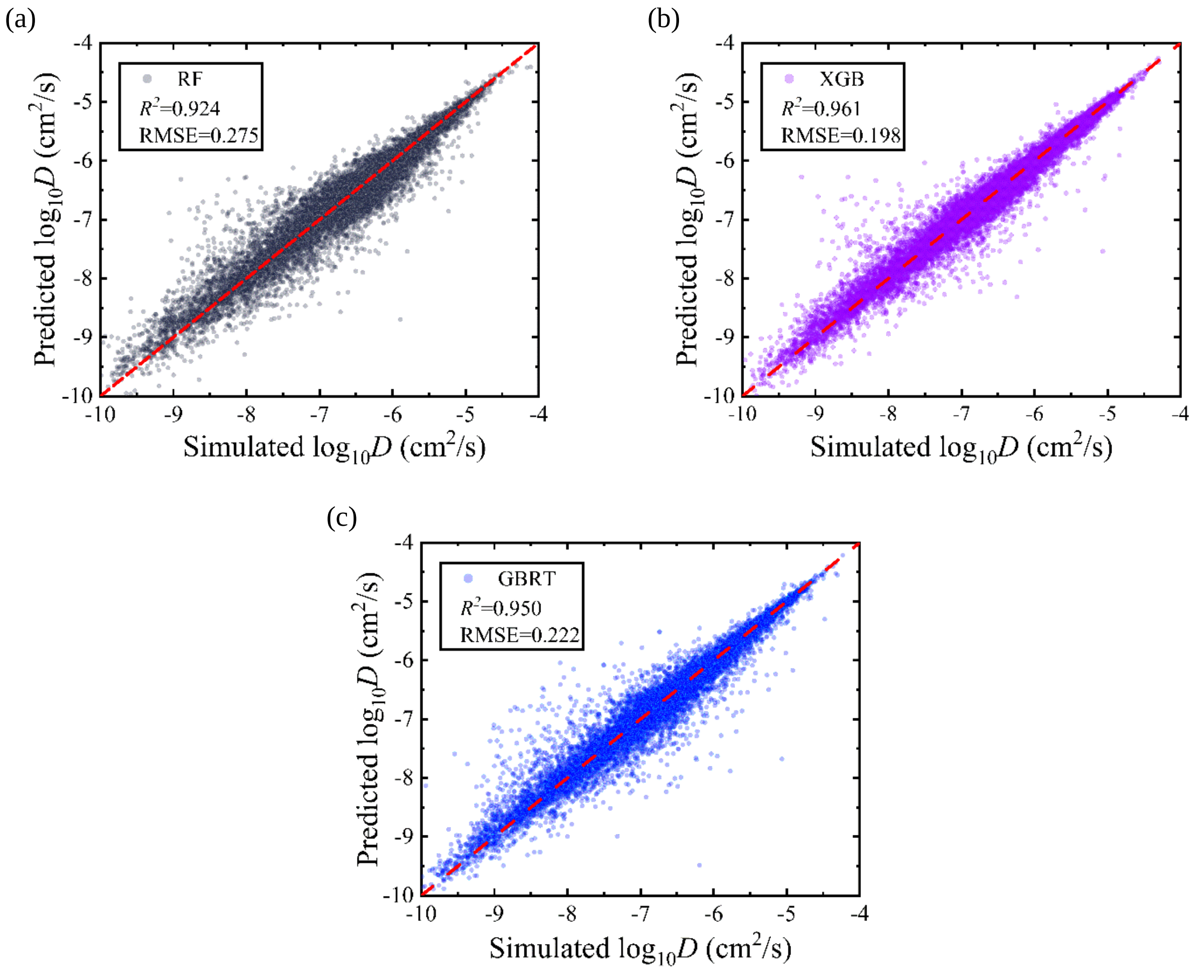

Figure S12. Predicted results of D by RF, XGB, and GBRT ML algorithm models *versus* simulated results of CoRE-MOFs on the testing set.

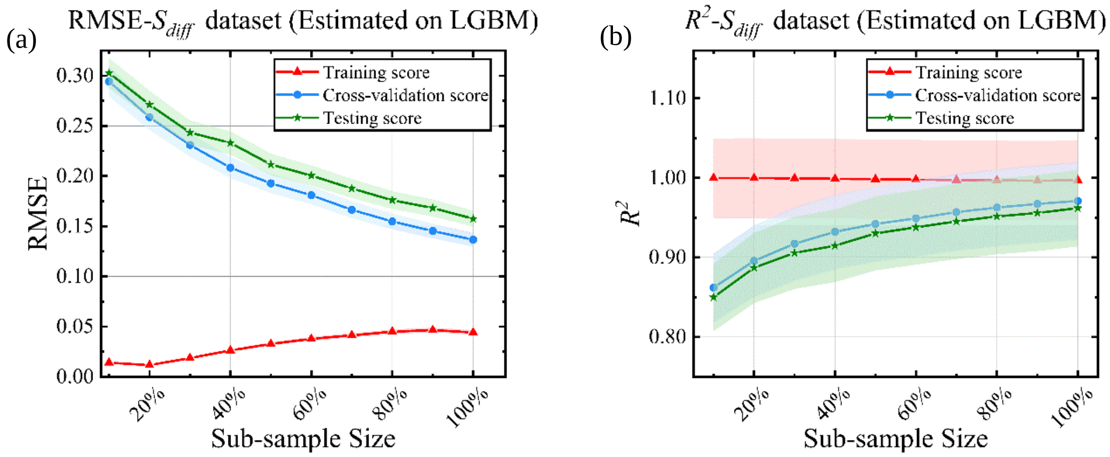

Figure S13. The performance of LGBM model for predicting S_{diff} under different sample sizes. (a) R^2 as a function of the sample-size percentage used for training LGBM model, (b) RMSE as a function of the sample-size percentage used for training LGBM model. The line in the graph shows the mean of 10-fold cross-validation. The shaded portion of each line in (a) and (b) represents the error band (±0.05).

Section S5. Analysis of the relative importance of features

In this work, predictions from an ML model were interpreted using TreeExplainer in SHAP (Shapley Additive explanation). The LGBM model and SHAP values are combined to evaluate each feature's relative importance (degree of influence on model output). The mean of absolute SHAP value per feature across the dataset represents the importance of feature influence. The results are shown in Tables S8 and S9.

Table S8. Importance ranking of features (Based on *D*)

No.	1	2	3	4	5	6	7	8	9
Feature	PLD	Dia_i	Pol_i	VSA	ϕ	LCD	Qua_i	ρ	Dip_i
Importance (%)	47.02	31.77	16.99	16.21	8.35	7.65	4.87	4.68	0.96

Table S9. Importance ranking of features (Based on S_{diff})

No.	1	2	3	4	5	6	7	8	9	10	11	12	13
Feature	ΔPol	PLD	Dia_i	VSA	ΔDia	Pol_i	LCD	ϕ	ρ	ΔQua	ΔDip	Qua_i	Dip_i
Importance (%)	38.78	12.44	11.53	10.36	9.71	6.15	6.15	4.23	3.51	2.13	0.79	0.66	0.09

Shapley additive explanation

In this work, SHAP (Shapley Additive explanations) is used to explain the importance and role of different predictors in the analysis. As a game theory approach, SHAP interprets the predicted values of the model as the sum of the imputed values for each input feature, and when approximating the original model f for a particular input x, the explanation's attribution values ϕ_i for each feature *i* should sum up to the output *f(x)*, represented by equation S3:

$$f(x)=\phi_0(f)+\sum_{i=1}^{M}\phi_i(f,x) \qquad \text{(S3).}$$

Where the sum of the feature attributes$\varphi_i(f,x)$ matches the output *f(x)* of the original model, M is the total number of input features, ϕ_0 represents the expected value when all inputs are missing, and ϕ_i is a measure of the contribution of a given feature *i* to the prediction. According to game theory, the Shapley value is the only criterion that satisfies local accuracy, missing, and consistency. They are also very intuitive because they use the same units as the model output (*D* or S_{diff} in this work). SHAP value is the Shapley value of a conditional expectation function *f(x),* which can be derived from equation S4:

$$\phi_i=\sum_{R\in\mathcal{R}}\frac{1}{M!}[f_x(P_i^R\cup i)-f_x(P_i^R)] \qquad \text{(S4),}$$

where $\mathcal{R}$ is the set of all feature orderings, P_i^R is the set of all features that come before feature *i* in ordering R, and M is the number of input features for the model. For tree-based models, the study's algorithm,

TreeExplainer, built by Lundberg et al.,[21] effectively computes this value. The TreeExplainer SHAP value is predicted individually for each sample in the dataset, and then the results of all predictions are plotted to show the global interpretation. To further understand the local contribution of features, it can be investigated by pairwise interactive SHAP interaction values of any two features *i* and *j*. SHAP interaction values $\phi_{i,j}$ can be obtained from equation S5,

$$\phi_{i,j}(f,x)=\sum_{S\subset M\{i,j\}}\frac{|S|!(M-|S|-2)!}{2(M-1)!}\nabla_{ij}(f,x,S) \qquad (S5).$$

When $i\neq j$， and

$$\nabla_{ij}(f,x,S)=f_x(S\cup i,j)-f_x(S\cup i)-f_x(S\cup j)+f_x(S) \qquad (S6),$$

$$\phi_{i,i}(f,x)=\phi_i(f,x)-\sum_{j\neq i}^{M}\phi_{i,j}(f,x) \qquad (S7).$$

When we set $\phi_{0,0}(f,x)=f_x(\varnothing)$,$\phi(f,x)$ sums to the output of the model:

$$\sum_{i=1}^{M}\sum_{i=1}^{M}\phi_i(f,x)=f(x) \qquad (S8).$$

In equation S5, SHAP interaction values of feature *i* and feature *j* are equally distributed between each feature, so $\phi_{i,j}(f,x)=\varphi_{j,i}(f,x)$, and the overall interaction effect is $\phi_{i,j}(f,x)+\phi_{j,i}(f,x)$. The residual effect can be defined as the difference between the SHAP value of the feature and the non-diagonal SHAP interaction value, as calculated by equation S6. SHAP interaction values have properties similar to SHAP values.[22] Python implementations of these metrics are available online at https://github.com/ suinleelab/treeexplainer-study.

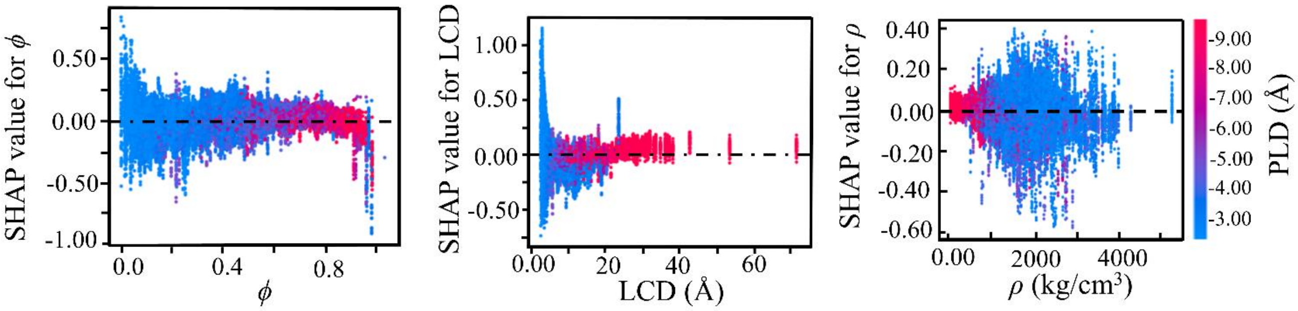

Figure S14. SHAP dependence plot of ϕ、LCD、ρ and PLD on making predictions by the LGBM model for S_{diff}

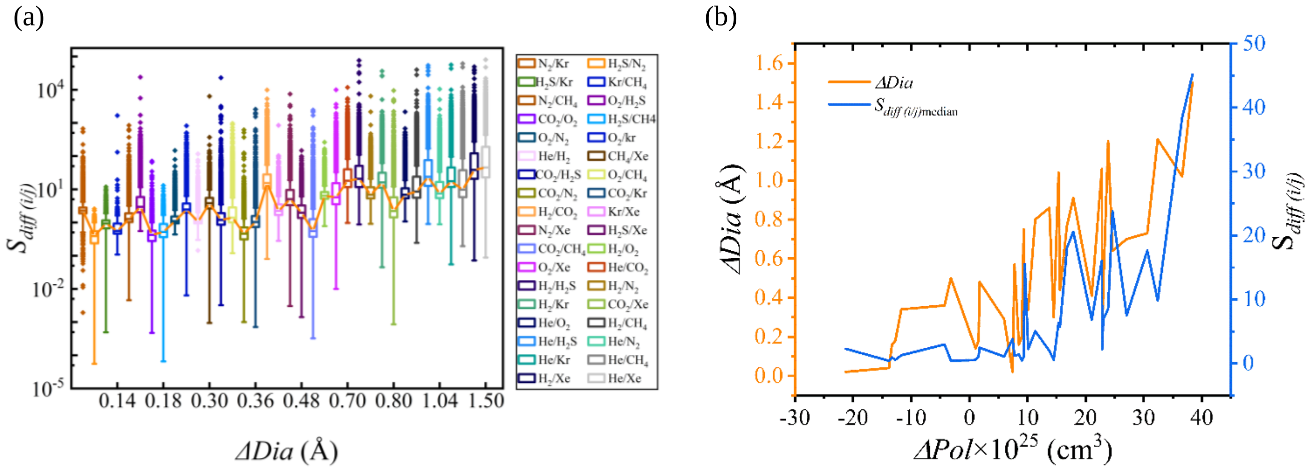

Figure S15. The relationship between *ΔDia* and diffusion selectivity (S_{diff}). (a) Box and whisker diagrams for the heat of S_{diff} of each molecule pair in 6013 MOFs. The center line of each box represents the median of data and the square symbol is centered on the mean of data. The upper and lower horizontal lines provide the minimum value (Q1-1.5×IQR) and maximum value (Q3+1.5×IQR), respectively, where IQR equals Q3-Q1. Outliers beyond this range are shown as individual data points. (b) Double y-axis line chart, on the right side of the y-axis represents *ΔDia* and on the left side of the y-axis represents (a) 6013MOFs of S_{diff} of median, the horizontal coordinate is *ΔPol* of 36 molecular mixtures, from big to small order.

The trajectories of Kr and Xe in MOFs

In statistical mechanics, the mean squared displacement (MSD, also mean square displacement or average squared displacement) is the most common measure of the spatial extent of random motion. Einstein showed that the mean square displacement of a particle in a liquid grows linearly with time. For a stable solid, this quantity will stay stable over the course of the simulation, and MSD was calculated by Einstein Eq. S9

$$D = \frac{1}{2n}\lim_{t\to\infty}\frac{\langle (r_t - r_0)^2 \rangle}{t} \qquad \text{(S9)}$$

In this work, the coefficient in the denominator n is 4, because the gas molecules diffused in the 3D x -y -z plane, and we focused on simulations with 10 gas molecules in FAPYEA, BUSQIQ, FUDQIF, ELUQIM06.

Table S10. Interaction between MOFs and Kr, Xe.

MOF-Molecule	Average Interaction Energy [kJ/mol]	ΔE_{tot} [kJ/mol]
FAPYEA-Xe	30.31	8.14
FAPYEA-Kr	22.17	
BUSQIQ-Xe	41.77	4.23
BUSQIQ-Kr	37.54	
FUDQIF-Xe	44.91	9.51
FUDQIF-Kr	35.40	
ELUQIM06-Xe	37.22	7.08
ELUQIM06-Kr	30.14	

*ΔE_{tot} represents the difference in the interaction forces between Xe and Kr and MOF, $\Delta E_{tot} = E_{MOF\text{-}Xe} - E_{MOF\text{-}Kr}$.

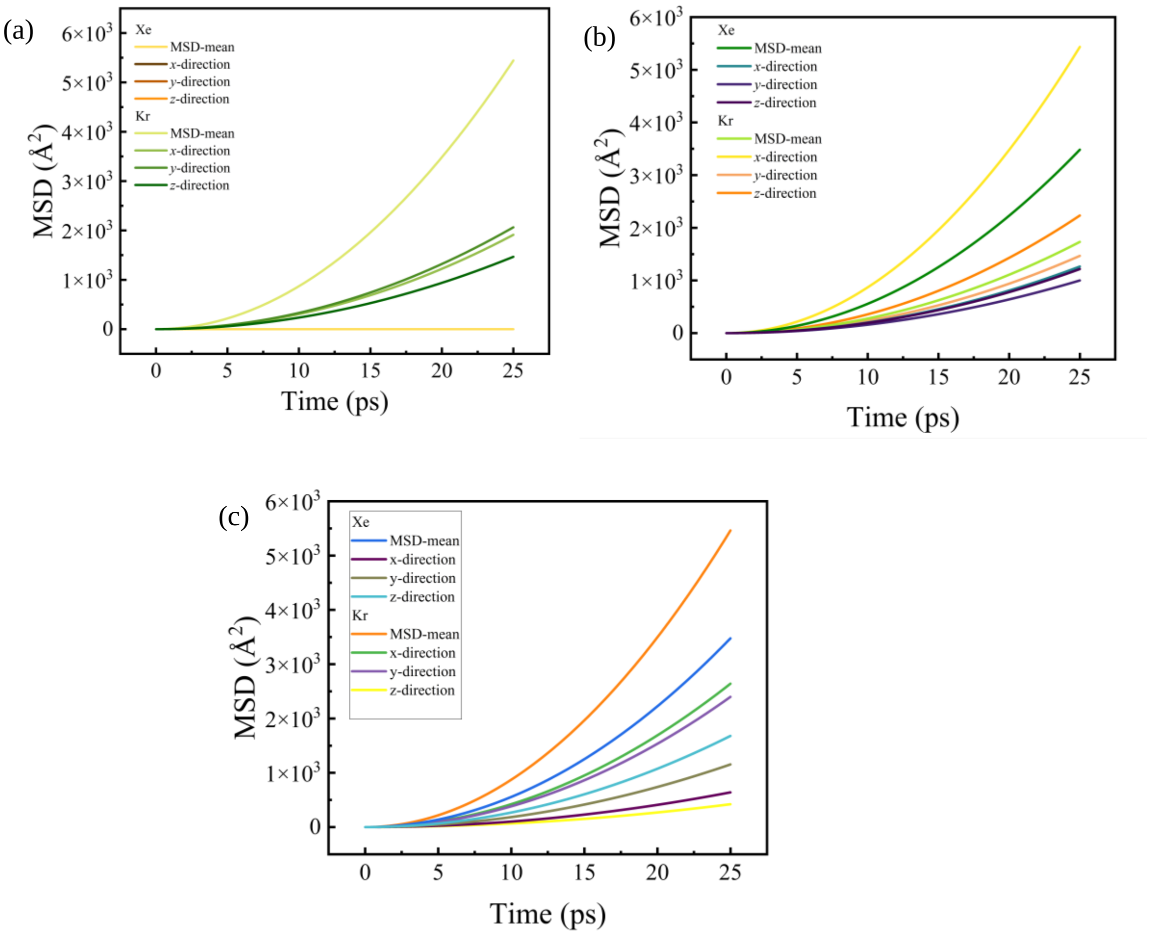

Figure S16. Total MSDs and the x-, y-, and z-components of MSDs of Kr and Xe in MOFs. (a) BUSQIQ, (b) FUDQIF, (c) ELUQIM06.

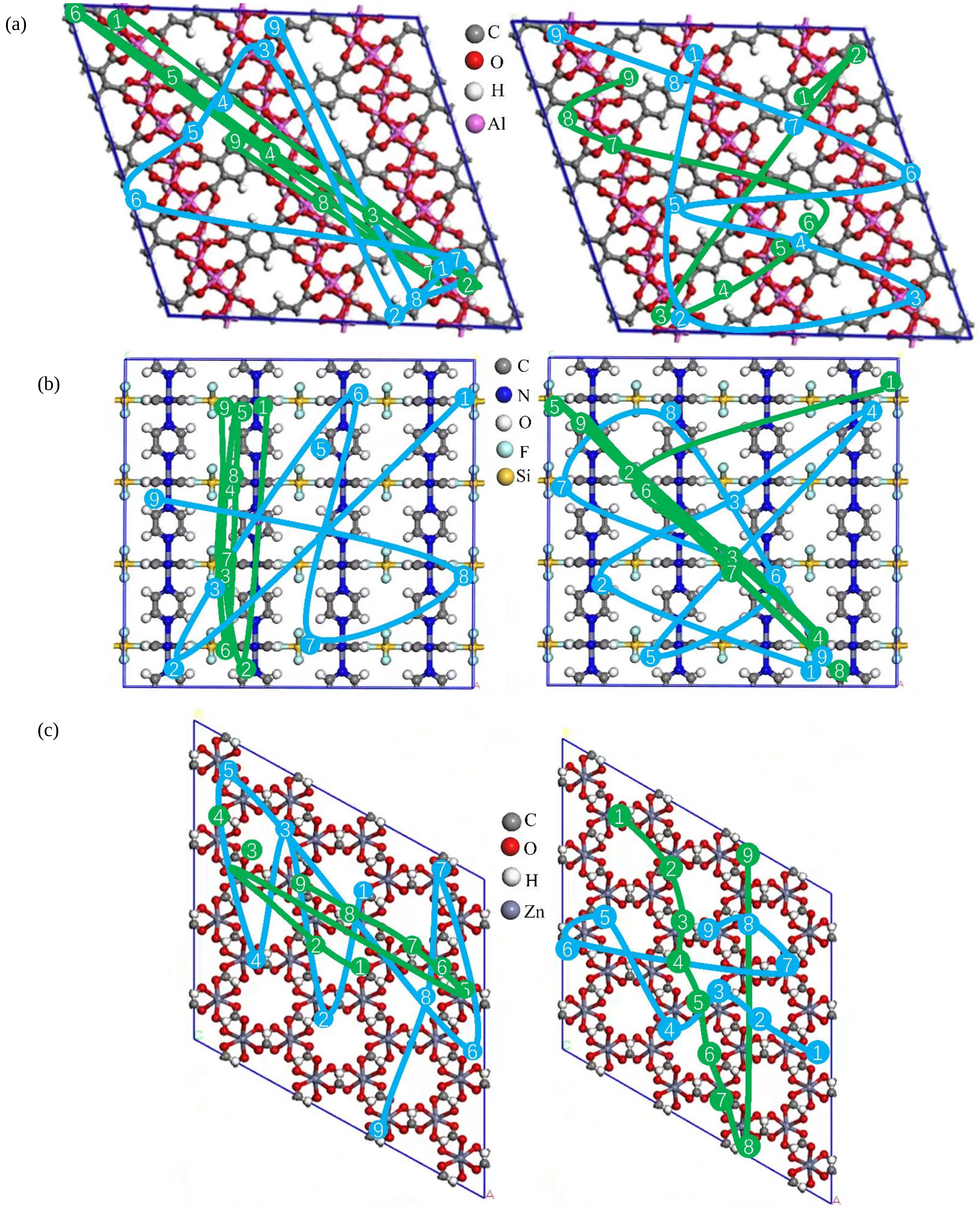

Figure S17. Diffusion pathways of two Xe and Kr molecules in BUSQIQ, FUDQIF, and ELUQIM06, respectively (Xe, green; Kr, blue

Section S6. Prediction of diffusivity of other gases (Extrapolation)

Table S11. Benchmark of D_i and $S_{diff(i/j)}$ for Additional Extrapolated eight mixtures

Gas mixture *i/j*	D_i	$S_{diff\ (i/j)}$	Gas mixture *i/j**	D_i	$S_{diff\ (i/j)}$
He/C_2H_6	9.90E-06	16	C_2H_6/ H_2S	3.50E-07	90
H_2/C_2H_6	1.00E-05	10	N_2/C_2H_6	6.00E-08	6
CO_2/C_2H_6	1.50E-07	6	C_2H_6/ Kr	7.00E-07	5
O_2/C_2H_6	3.00E-09	5	C_2H_6/ CH_4	2.00E-07	18

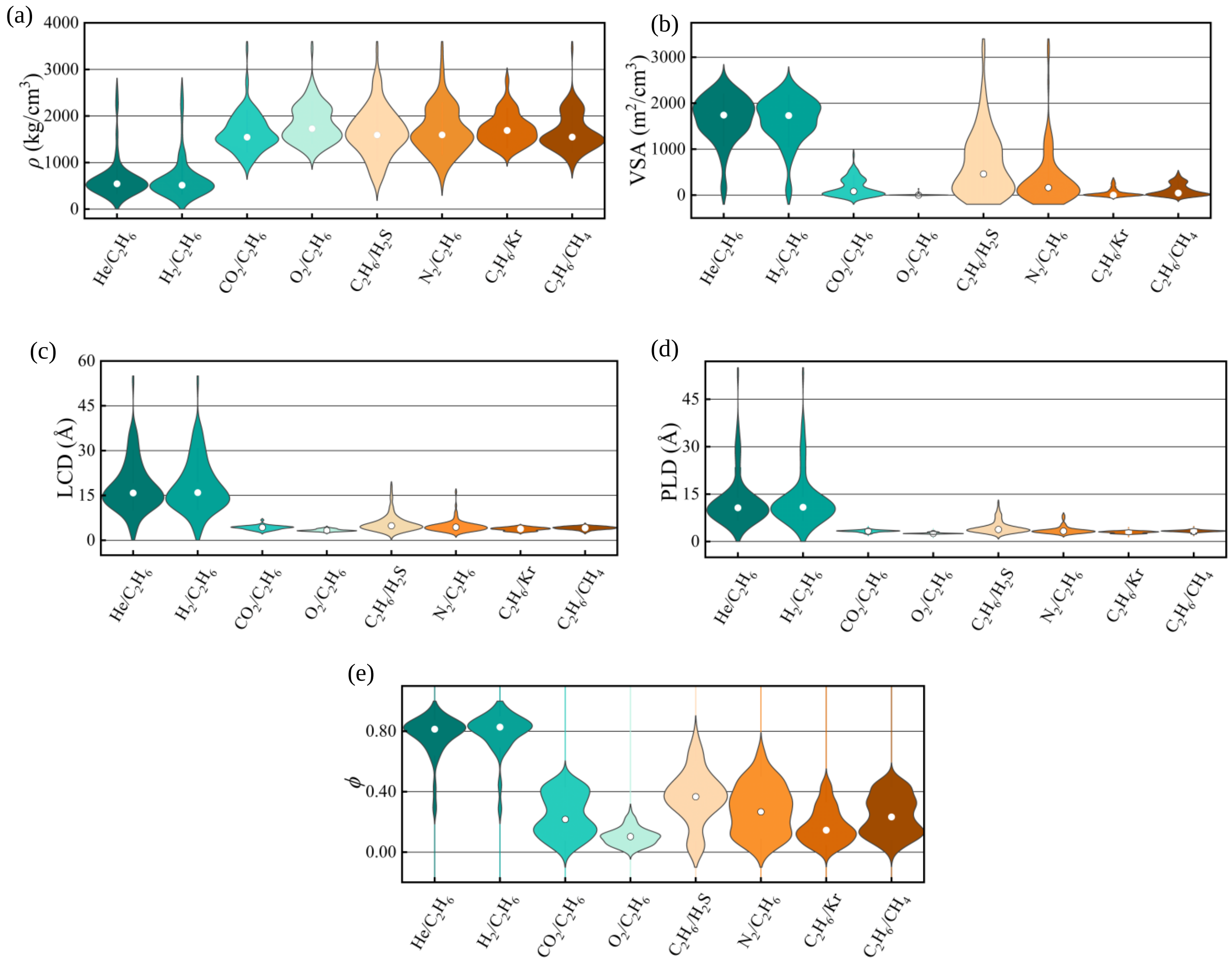

Figure S18. Approximate distribution of ρ, VSA, LCD, PLD, and ϕ of about TOP 100 MOFs for 8 kinds of binary gas mixtures for prediction. The colors represent different gas mixtures, and the white ball represents the median.

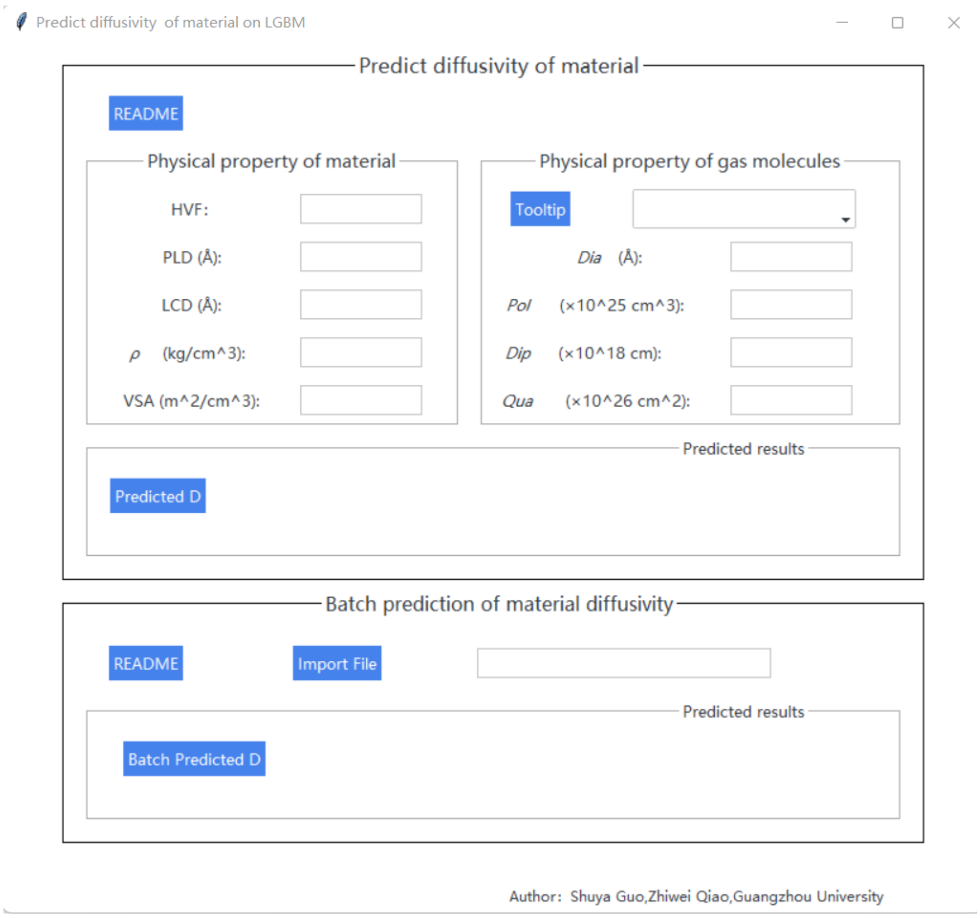

Figure S19. The main interface of an interactive desktop application for predicting the diffusivity of material.

Table S12. The version information of tool package for the interactive desktop application design

Package	Version
sklearn	1.02
pandas	1.3.5
lightgbm	3.3.2
joblib	1.1.0
ttkbootstrap	1.9.0
pyinstaller	5.4.1

Notes: To design the interactive desktop application, we utilize ttkbootstrap, a common Python GUI module, and pyinstaller to package exe files.

Section S7. Top-performing MOFs

Table S13. Benchmark of D_i and $S_{diff\,(i/j)}$ for thirty-six gas mixtures

Gas mixture *i/j*	D_i	$S_{diff\,(i/j)}$	Gas mixture *i/j**	D_i	$S_{diff\,(i/j)}$
He/H_2	1.00E-09	9.7	CO_2/Kr	1.00E-07	10
He/CO_2	3.50E-06	140	CO_2/CH_4	9.00E-08	10
He/O_2	8.80E-07	70	CO_2/Xe	1.20E-07	60
He/H_2S	2.00E-06	550	O_2/H_2S	1.00E-06	6
He/N_2	9.50E-07	200	O_2/N_2	4.00E-08	5
He/Kr	1.40E-06	500	O_2/Kr	4.00E-07	5.5
He/CH_4	9.50E-07	1000	O_2/CH_4	1.00E-07	26
He/Xe	1.60E-06	1700	O_2/Xe	1.00E-06	8
H_2/CO_2	2.10E-06	300	H_2S/N_2	3.00E-09	1
H_2/O_2	3.00E-07	25	H_2S/Kr	3.00E-07	1.5
H_2/H_2S	1.70E-06	400	H_2S/CH_4	1.00E-09	1.9
H_2/N_2	7.00E-07	70	H_2S/Xe	1.00E-07	13
H_2/Kr	1.00E-06	270	N_2/Kr	1.00E-07	7
H_2/CH_4	1.00E-06	300	N_2/CH_4	1.00E-07	5.5
H_2/Xe	1.60E-06	1000	N_2/Xe	7.00E-07	11
CO_2/O_2	1.20E-07	1	Kr/CH_4	1.50E-08	1.2
CO_2/H_2S	1.10E-07	10	Kr/Xe	1.00E-07	12
CO_2/N_2	8.50E-08	2	CH_4/Xe	5.00E-07	10

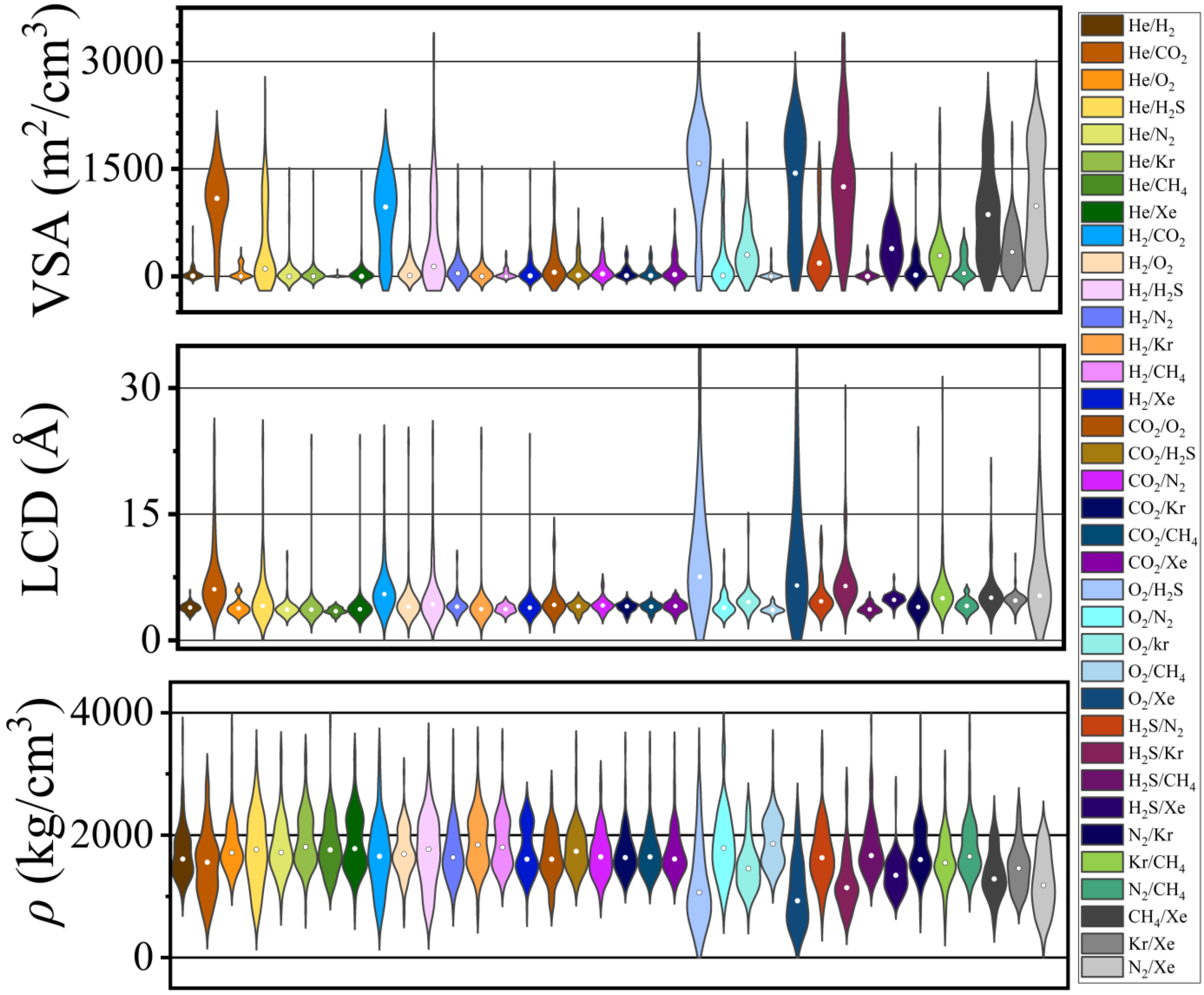

Figure S20. Approximate distribution of VSA, LCD, and ρ of about TOP 100 MOFs for 36 mixed gas pairs. The colors represent different gas mixtures, and the white ball represents the median.

Table S14. Top-performing MOFs for the separation of different gas mixtures

Gas mixture *i/j**	CSD	LCD [Å]	ϕ	PLD [Å]	ρ [kg/m^3]	D_i [cm^2/s]	D_j [cm^2/s]	$S_{diff(i/j)}$
He/H_2	SODKON	3.19	0.02	2.70	2696.92	2.17E-06	1.85E-08	117.09
	OFIWIK	4.20	0.05	3.14	1866.86	1.10E-06	9.47E-09	116.31
	WIJDID	2.83	0.02	2.41	2689.07	5.20E-08	6.78E-10	76.71
He/CO_2	MOTMAK	5.72	0.56	5.33	997.47	7.60E-06	6.46E-10	11774.62
	JEDJUY	5.06	0.49	4.15	1669.62	6.22E-06	1.15E-09	5424.87
	PEJMOI	7.77	0.39	4.72	1789.18	4.63E-06	1.09E-09	4264.74
He/O_2	SODKON	3.19	0.02	2.70	2696.92	2.17E-06	1.00E-09	2168.42
	CAXPEY	3.46	0.04	2.47	1840.44	1.29E-06	1.89E-09	682.95
	XOMCIL	4.28	0.19	2.55	3284.05	1.73E-06	3.26E-09	530.26
He/H_2S	ELUQIM06	2.89	0.04	2.41	1779.43	9.74E-06	1.80E-10	54199.91
	FAPYEA04	2.47	0.00	2.40	1583.54	2.42E-06	5.22E-11	46430.63
	ELUQIM04	2.92	0.04	2.44	1764.65	9.03E-06	2.44E-10	36973.99
He/N_2	ELUQIM06	2.89	0.04	2.41	1779.43	9.74E-06	9.25E-10	10527.88
	SODKON	3.19	0.02	2.70	2696.92	2.17E-06	4.28E-10	5071.94
	ELUQIM05	2.90	0.04	2.43	1773.83	8.42E-06	3.25E-09	2587.65
He/Kr	ELUQIM04	2.92	0.04	2.44	1764.65	9.03E-06	1.60E-10	56276.65
	ELUQIM05	2.90	0.04	2.43	1773.83	8.42E-06	1.93E-10	43522.95
	ELUQIM06	2.89	0.04	2.41	1779.43	9.74E-06	2.24E-10	43520.45
He/CH_4	ELUQIM04	2.92	0.04	2.44	1764.65	9.03E-06	1.47E-10	61406.36
	ELUQIM05	2.90	0.04	2.43	1773.83	8.42E-06	1.76E-10	47815.36
	ELUQIM06	2.89	0.04	2.41	1779.43	9.74E-06	8.11E-10	12015.02
He/Xe	ELUQIM04	2.92	0.04	2.44	1764.65	9.03E-06	1.12E-10	80818.96
	ELUQIM06	2.89	0.04	2.41	1779.43	9.74E-06	1.21E-10	80170.92
	ELUQIM05	2.90	0.04	2.43	1773.83	8.42E-06	1.76E-10	47914.49
H_2/CO_2	MOTMAK	5.72	0.56	5.33	997.47	6.38E-06	6.46E-10	9884.84
	JEDJUY	5.06	0.49	4.15	1669.62	6.07E-06	1.15E-09	5296.35
	HUCBAJ	5.86	0.58	3.90	1200.04	4.59E-06	1.21E-09	3802.41
H_2/O_2	ELUQIM06	2.89	0.04	2.41	1779.43	5.97E-06	2.28E-08	262.27
	VAZLUF	3.86	0.21	2.54	2122.82	1.10E-06	6.58E-09	166.62
	VAZMIU	3.77	0.21	2.44	2281.22	7.18E-07	4.82E-09	148.92
H_2/H_2S	FAPYEA	2.53	0.00	2.46	1582.41	6.39E-06	8.35E-11	76535.31
	FAPYEA04	2.47	0.00	2.40	1583.54	2.83E-06	5.22E-11	54232.60
	ELUQIM06	2.89	0.04	2.41	1779.43	5.97E-06	1.80E-10	33212.52
H_2/N_2	ELUQIM06	2.89	0.04	2.41	1779.43	5.97E-06	9.25E-10	6451.26
	ELUQIM05	2.90	0.04	2.43	1773.83	7.08E-06	3.25E-09	2174.07
	PEJMOI	7.77	0.39	4.72	1789.18	2.40E-06	2.79E-09	862.22
H_2/kr	ELUQIM05	2.90	0.04	2.43	1773.83	7.08E-06	1.93E-10	36566.71
	ELUQIM06	2.89	0.04	2.41	1779.43	5.97E-06	2.24E-10	26668.38
	ELUQIM04	2.92	0.04	2.44	1764.65	4.14E-06	1.60E-10	25796.85
H_2/CH_4	ELUQIM05	2.90	0.04	2.43	1773.83	7.08E-06	1.76E-10	40173.07
	ELUQIM04	2.92	0.04	2.44	1764.65	4.14E-06	1.47E-10	28148.27
	FAPYEA04	2.47	0.00	2.40	1583.54	2.83E-06	3.01E-10	9397.11
H_2/Xe	ELUQIM06	2.89	0.04	2.41	1779.43	5.97E-06	1.21E-10	49127.00
	ELUQIM05	2.90	0.04	2.43	1773.83	7.08E-06	1.76E-10	40256.35

	ELUQIM04	2.92	0.04	2.44	1764.65	4.14E-06	1.12E-10	37046.88
CO_2/O_2	HIQPEE	3.84	0.15	3.12	1440.14	1.24E-06	1.75E-08	70.88
	HIQPII	3.87	0.15	3.12	1472.40	1.13E-06	1.72E-08	65.47
	SAPJIE	4.08	0.07	3.02	1760.46	7.14E-07	2.74E-08	26.10
CO_2/H_2S	FAPYEA	2.53	0.00	2.46	1582.41	1.92E-06	8.35E-11	22922.93
	ELUQIM06	2.89	0.04	2.41	1779.43	5.71E-07	1.80E-10	3174.50
	FAPYEA04	2.47	0.00	2.40	1583.54	1.49E-07	5.22E-11	2850.26
CO_2/N_2	ELUQIM06	2.89	0.04	2.41	1779.43	5.71E-07	9.25E-10	616.62
	HIQPEE	3.84	0.15	3.12	1440.14	1.24E-06	2.43E-09	512.35
	HIQPII	3.87	0.15	3.12	1472.40	1.13E-06	3.03E-09	371.89
CO_2/kr	XEKDUO	2.98	0.02	2.75	1903.55	1.70E-07	6.57E-11	2593.23
	ELUQIM06	2.89	0.04	2.41	1779.43	5.71E-07	2.24E-10	2549.00
	ELUQIM05	2.90	0.04	2.43	1773.83	3.91E-07	1.93E-10	2021.03
CO_2/CH_4	XEKDUO	2.98	0.02	2.75	1903.55	1.70E-07	6.92E-11	2463.01
	ELUQIM05	2.90	0.04	2.43	1773.83	3.91E-07	1.76E-10	2220.35
	HIQPEE	3.84	0.15	3.12	1440.14	1.24E-06	5.69E-10	2185.18
CO_2/Xe	FAPYEA	2.53	0.00	2.46	1582.41	1.92E-06	2.02E-10	9494.58
	HIQPEE	3.84	0.15	3.12	1440.14	1.24E-06	2.43E-10	5112.41
	XEKDUO	2.98	0.02	2.75	1903.55	1.70E-07	3.54E-11	4807.12
O_2/H_2S	FAPYEA	2.53	0.00	2.46	1582.41	2.01E-06	8.35E-11	24027.90
	ZEKRIS	5.65	0.54	4.82	1034.67	1.29E-06	1.90E-09	677.73
	JEDJUY	5.06	0.49	4.15	1669.62	1.12E-06	2.05E-09	546.31
O_2/N_2	PEJMOI	7.77	0.39	4.72	1789.18	4.13E-07	2.79E-09	148.16
	BEVQUP	2.88	0.14	2.45	1937.85	7.31E-08	9.20E-10	79.39
	LISZOE	3.55	0.22	3.01	1535.50	1.49E-07	2.95E-09	50.55
O_2/Kr	HIWXER	3.23	0.14	2.68	2549.13	4.87E-07	1.89E-09	257.20
	PARFOF	2.77	0.05	2.46	1541.02	5.04E-07	2.13E-09	236.88
	HIWXER01	3.29	0.13	2.76	2533.00	4.47E-07	2.40E-09	185.92
O_2/CH_4	FAPYEA04	2.47	0.00	2.40	1583.54	2.92E-07	3.01E-10	968.16
	ELUQIM05	2.90	0.04	2.43	1773.83	1.58E-07	1.76E-10	898.78
	GUXQAS	2.79	0.02	2.52	1598.28	1.16E-07	1.30E-10	893.53
O_2/Xe	FAPYEA	2.53	0.00	2.46	1582.41	2.01E-06	2.02E-10	9952.26
	AHOKIR01	4.31	0.46	3.47	1927.02	1.55E-06	1.90E-09	817.06
	SADMIV	3.02	0.13	2.79	1566.89	1.46E-06	5.10E-09	286.54
H_2S/N_2	WEXNIW	3.58	0.21	2.65	2137.25	4.73E-09	1.82E-09	2.60
	MUPYIG	3.47	0.12	2.40	2412.55	3.72E-09	1.63E-09	2.29
	ZEJCUO	3.46	0.19	2.60	2142.29	5.56E-09	3.12E-09	1.78
H_2S/kr	PUWCUF	5.63	0.59	3.93	1205.85	3.60E-07	9.82E-08	3.67
	SADMIV	3.02	0.13	2.79	1566.89	3.72E-07	1.21E-07	3.07
	ja205976v	11.09	0.68	6.66	1149.65	1.73E-06	6.18E-07	2.79
H_2S/CH_4	GUXQAS	2.79	0.02	2.52	1598.28	1.70E-09	1.30E-10	13.04
	RUPZIM	3.48	0.11	3.25	1549.49	1.51E-07	1.39E-08	10.87
	GUXPUL	2.79	0.02	2.58	1595.02	1.18E-09	1.10E-10	10.70
H_2S/Xe	RUPZIM	3.48	0.11	3.25	1549.49	1.51E-07	1.02E-09	148.04
	SADMIV	3.02	0.13	2.79	1566.89	3.72E-07	5.10E-09	72.91
	IKOSUW	4.08	0.35	3.55	1120.10	2.52E-07	4.47E-09	56.33
N_2/kr	PARFOF	2.77	0.05	2.46	1541.02	4.78E-07	2.13E-09	224.91
	HIWXER	3.23	0.14	2.68	2549.13	2.45E-07	1.89E-09	129.49

	FAPYEA04	2.47	0.00	2.40	1583.54	1.11E-07	1.33E-09	83.75
	FAPYEA04	2.47	0.00	2.40	1583.54	1.11E-07	3.01E-10	369.05
N_2/CH4	PARFOF	2.77	0.05	2.46	1541.02	4.78E-07	2.16E-09	221.18
	HIWXER01	3.29	0.13	2.76	2533.00	1.80E-07	1.27E-09	141.34
	FAPYEA	2.53	0.00	2.46	1582.41	1.51E-06	2.02E-10	7462.93
N_2/Xe	KUXTUT	3.39	0.09	3.18	1534.92	9.63E-07	3.09E-10	3119.67
	IYIGEC	3.42	0.07	3.18	2163.70	9.71E-07	5.91E-10	1642.40
	POZHUI	9.39	0.99	3.79	1305.42	1.38E-05	1.05E-07	132.05
Kr/CH_4	ja406030p	11.78	0.92	6.59	794.73	6.37E-06	3.28E-07	19.45
	ja406030p	11.03	0.92	8.49	735.76	5.38E-06	2.80E-07	19.24
	FAPYEA	2.53	0.00	2.46	1582.41	1.70E-07	2.02E-10	843.55
Kr/Xe	XAXQEU	3.64	0.23	3.21	1726.54	1.21E-07	1.28E-09	94.83
	BUSQIQ	4.44	0.61	3.73	1569.82	9.77E-07	1.37E-08	71.44
	FAPYEA	2.53	0.00	2.46	1582.41	1.28E-06	2.02E-10	6339.05
CH_4/Xe	FUDQIF	4.38	0.37	3.86	1573.76	8.92E-06	4.16E-08	214.41
	MAZTIR	3.47	0.29	3.22	2204.28	1.70E-06	1.44E-08	117.64

Table S15. Top-performing MOFs for the catalyst of CO_2 methanation

No.	CSD code	OMS	PLD [Å]	LCD [Å]	ϕ	VSA [m^2/cm^3]	ρ [kg/m^3]	D_{CO_2} [cm^2/s]
1	QIYDIN	Cu	15.0225	29.975	0.94	1362.178	197.64	2.6426×10^{-6}
2	CIGXIA	Fe	11.8329	28.497	0.78	1329.749	684.424	2.4544×10^{-6}
3	NIBJAK	Cu	17.5518	32.002	0.94	1235.175	223.43	2.1678×10^{-6}
4	UDEMEW	Cr	11.695	28.243	0.77	1342.323	681.201	2.0776×10^{-6}
5	XOGZEZ	Si	11.161	27.809	0.83	1326.749	630.771	2.0492×10^{-6}
6	ADATEG	Cu	13.339	27.338	0.92	1525.721	238.773	1.8741×10^{-6}
7	DAWMUL	Cu	14.3252	28.65	0.93	1544.331	266.213	1.7698×10^{-6}
8	HEXVEM	Cu	15.9175	28.431	0.93	1391.354	251.731	1.675×10^{-6}
9	GUPBEZ02	Cu	10.8478	23.871	0.76	1568.637	911.24	1.6733×10^{-6}
10	GAQYIH	Cd	8.96976	20.258	0.84	2104.531	559.91	1.6569×10^{-6}
11	ADUROI	Zn	17.1241	25.686	0.85	1255.323	402.545	1.6032×10^{-6}
12	ADASAB	Cu	13.4901	22.822	0.88	1654.329	343.939	1.5877×10^{-6}
13	NIBHOW	Cu	14.8846	27.512	0.92	1447.975	279.591	1.5653×10^{-6}
14	QOWRAV01	Cu	8.5601	20.153	0.9	1900.429	342.058	1.3205×10^{-6}
15	XAFFUH	Cu	19.638	23.728	0.9	1734.826	329.161	1.3018×10^{-6}
16	BAZFUF	Cu	8.58384	20.239	0.91	1888.591	340.049	1.284×10^{-6}
17	HABQUY	Cu	12.0883	25.715	0.91	1670.99	289.448	1.2826×10^{-6}
18	SEMNIJ	Cu	13.5802	31.593	0.94	1387.093	222.123	1.2652×10^{-6}
19	DITJIB	Hf	9.01147	20.363	0.87	1820.958	521.413	1.1979×10^{-6}
20	GAGZEV	Cu	11.5377	28.665	0.91	1623.29	279.145	1.1637×10^{-6}
21	KOZQIB	Cu	12.236	28.541	0.93	1473.495	302.86	1.117×10^{-6}
22	QIYDAF01	Cu	13.5793	22.001	0.9	1721.174	303.246	1.1104×10^{-6}
23	ADATIK	Cu	12.1743	24.551	0.89	1729.693	377.573	1.062×10^{-6}
24	CAVPUM	Zn	7.78037	20.428	0.85	1927.625	407.431	1.0578×10^{-6}
25	PODKUQ	Cu	8.92625	22.319	0.89	2029.165	425.76	9.9924×10^{-7}
26	HABRAF	Cu	9.03547	24.3	0.89	1852.441	382.336	9.6525×10^{-7}
27	ADATAC	Zn	10.2764	26.335	0.9	1747.078	337.249	9.5769×10^{-7}
28	SEMNEF	Cu	12.9373	30.847	0.93	1452.196	237.227	9.5735×10^{-7}
29	UBULIO	Zn	22.8992	24.232	0.85	1768.302	438.033	9.3847×10^{-7}
30	LURRIA	Cu	9.74126	22.373	0.91	1865.766	406.537	9.1277×10^{-7}
31	VETTIZ	Fe	12.8543	21.625	0.83	1553.373	537.59	8.9092×10^{-7}
32	KOZQEX	Cu	6.86083	22.424	0.88	1954.897	430.909	8.8762×10^{-7}
33	MEHMET	Cu	9.06966	21.825	0.88	1877.565	408.792	8.8472×10^{-7}
34	QIYDAF	Cu	8.76388	22.98	0.84	1766.739	403.146	8.7235×10^{-7}
35	TOVKOG	Fe	13.1458	22.842	0.9	1546.859	331.596	8.6438×10^{-7}
36	BIWSEG	Cu	11.97	29.735	0.99	1334.81	466.935	8.5299×10^{-7}
37	BAZGEQ	Cu	13.9632	27.247	0.88	1555.041	334.889	8.1579×10^{-7}
38	VETTAR	Ni	12.0081	20.435	0.8	1649.689	585.001	7.465×10^{-7}
39	ADASEF	Cu	10.9231	21.624	0.89	1830.23	435.603	6.9283×10^{-7}
40	VETSOE	Mn	12.4603	20.9	0.78	1586.524	550.117	6.69×10^{-7}
41	VETSUK	Co	12.0386	20.384	0.79	1622.684	623.875	6.6655×10^{-7}
42	YUSWEP	Cu	13.0059	37.318	0.87	1669.482	403.885	6.418×10^{-7}
43	LUKLIN	Cu	9.81612	20.058	0.88	1921.871	466.063	6.2834×10^{-7}
44	LUXLOG	Ni	8.74134	21.379	0.85	1914.22	420.407	6.2068×10^{-7}

45	TOFTIT	Cu	6.28286	20.163	0.82	2095.607	598.398	6.0938×10^{-7}
46	LEHXUT	Cu	9.11981	24.949	0.87	1867.997	407.167	6.0206×10^{-7}
47	QIYDEJ	Cu	11.5568	22.49	0.83	1808.655	431.42	5.7945×10^{-7}
48	NIMQEH	Cu	7.78548	23.252	0.86	1877.343	457.818	5.7518×10^{-7}
49	SOKXOH	Cu,Co	14.7443	22.967	0.89	1845.117	423.624	5.3583×10^{-7}
50	IZEPEJ	Cr	6.75259	23.874	0.81	1749.013	545.418	5.2756×10^{-7}
51	FIZPOV	Cu	9.90922	20.376	0.87	1846.794	515.247	5.2384×10^{-7}
52	RAVWES	Mg	23.6272	24.441	0.82	1223.714	439.053	5.2029×10^{-7}
53	BEDYEQ	Zr,Fe	31.4905	33.059	0.86	1354.703	462.44	5.2018×10^{-7}
54	PELRIJ	Cu	10.3834	21.556	0.83	1725.612	517.074	5.1395×10^{-7}
55	VEGCAN	Hf	8.97126	26.231	0.85	1727.966	749.46	5.1195×10^{-7}
56	TOVGAO	Ni	8.7248	21.396	0.85	1913.601	420.407	5.1005×10^{-7}
57	WITKIV	Zn	11.4837	20.461	0.78	1564.69	586.911	4.9938×10^{-7}
58	OCUNAC	Cr	14.0466	36.151	0.84	1359.353	427.516	4.6833×10^{-7}
59	VEGBUG	Zr	9.06745	26.295	0.85	1724.644	576.597	4.3296×10^{-7}
60	BINSAU	Mn	9.52705	21.88	0.83	1824.056	471.568	3.3687×10^{-7}
61	XOFGAC	Eu	4.16314	20.277	0.71	1606.002	983.667	2.6158×10^{-7}
62	IZEPAF	Cr	7.53543	24.1	0.8	1857.71	570.647	2.2382×10^{-7}
63	GUVYON	Zr	3.81745	23.564	0.98	1410.95	771.285	1.861×10^{-8}
64	NUSVEE	Zn	3.81745	23.564	0.97	1410.011	771.285	1.4287×10^{-8}
65	YENLUA	Cu,Na	18.9061	21.492	0.63	1211.955	794.829	1.4094×10^{-8}

References

[1]. Rappi, A. K.; Casewit, C. J.; Colwell, K. S.; III, W. A. G.; Skid, W. M., UFF, a Full Periodic Table Force Field for Molecular Mechanics and Molecular Dynamics Simulations. J. Am. Chem. Soc. 1992, 114 (25), 10024-10039.

[2]. Martin, M. G.; Siepmann, J. I., Transferable Potentials for Phase Equilibria. 1. United-Atom Description of n-Alkanes. J. Phys. Chem. B 1998, 102 (14), 2569-2577.

[3]. Shah, M. S.; Tsapatsis, M.; Siepmann, J. I., Development of the Transferable Potentials for Phase Equilibria Model for Hydrogen Sulfide. J. Phys. Chem. B 2015, 119 (23), 7041−7052.

[4]. Pillai, R. S.; Jobic, H.; Koza, M. M.; Nouar, F.; Serre, C.; Maurin, G.; Ramsahye, N. A., Diffusion of Carbon Dioxide and Nitrogen in the Small-Pore Titanium Bis(phosphonate) Metal-Organic Framework MIL-91 (Ti): A Combination of Quasielastic Neutron Scattering Measurements and Molecular Dynamics Simulations. Chemphyschem 2017, 18 (19), 2739-2746.

[5]. Chanajaree, R.; Sailuam, W.; Seehamart, K., Molecular Self-Diffusivity and Separation of CH_4/H_2S in Metal Organic Framework MIL-47(V). Micropor Mesopor Mat. 2022, 335, 111783.

[6]. Polat, H. M.; Zeeshan, M.; Uzun, A.; Keskin, S., Unlocking CO_2 Separation Performance of Ionic Liquid/CuBTC Composites: Combining Experiments with Molecular Simulations. Chem. Eng. J. 2019, 373, 1179-1189.

[7]. Gao, S.; Liu, Z.; Xu, S.; Zheng, A.; Wu, P.; Li, B.; Yuan, X.; Wei, Y.; Liu, Z., Cavity-Controlled Diffusion in 8-Membered Ring Molecular Sieve Catalysts for Shape Selective Strategy. J. Catal. 2019, 377, 51-62.

[8]. Zhang, C.; Lively, R. P.; Zhang, K.; Johnson, J. R.; Karvan, O.; Koros, W. J., Unexpected Molecular Sieving Properties of Zeolitic Imidazolate Framework-8. J. Phys. Chem. Lett. 2012, 3 (16), 2130−2134.

[9]. Daglar, H.; Keskin, S., Computational Screening of Metal-Organic Frameworks for Membrane-Based $CO_2/N_2/H_2O$ Separations: Best Materials for Flue Gas Separation. J. Phys. Chem. C 2018, 122 (30), 17347-17357.

[10]. Altintas, C.; Avci, G.; Daglar, H.; Gulcay, E.; Erucar, I.; Keskin, S., Computer Simulations of 4240 MOF Membranes for H_2/CH_4 Separations: Insights into Structure-Performance Relations. J. Mater. Chem. A 2018, 6 (14), 5836-5847.

[11]. Glover, J.; Besley, E., A High-Throughput Screening of Metal–Organic Framework Based Membranes for Biogas Upgrading. Faraday Discuss. 2021, 231, 235-257.

[12]. Altintas, C.; Keskin, S., Molecular Simulations of MOF Membranes and Performance Predictions of MOF/Polymer Mixed Matrix Membranes for CO_2/CH_4 Separations. ACS Sustain. Chem. Eng. 2019, 7 (2), 2739-2750.

[13]. Avci, G.; Velioglu, S.; Keskin, S., High-Throughput Screening of MOF Adsorbents and Membranes for H_2 Purification and CO2 Capture. ACS Appl. Mater. Interfaces 2018, 10 (39), 33693-33706.

[14]. Daglar, H.; Erucar, I.; Keskin, S., Exploring the Performance Limits of MOF/Polymer MMMs for O_2/N_2 Separation Using Computational Screening. J. Membr. Sci. 2021, 618.

[15]. Ramsahye, N. A.; Gao, J.; Jobic, H.; Llewellyn, P. L.; Yang, Q.; Wiersum, A. D.; Koza, M. M.; Guillerm, V.; Serre, C.; Zhong, C. L.; Maurin, G., Adsorption and Diffusion of Light Hydrocarbons in UiO-66(Zr): A Combination of Experimental and Modeling Tools. J. Phys. Chem. C 2014, 118 (47), 27470-27482.

[16]. Li, J. R.; Kuppler, R. J.; Zhou, H. C., Selective Gas Adsorption and Separation in Metal-Organic Frameworks. Chem. Soc. Rev. 2009, 38 (5), 1477-504.

[17]. Yin, F. F.; Shao, X. Y.; Zhao, L. J.; Li, X. P.; Zhou, J. Y.; Cheng, Y.; He, X. J.; Lei, S.; Li, J. G.; Wang, J. L., Predicting Prognosis of Endometrioid Endometrial Adenocarcinoma on the Basis of Gene Expression and Clinical Features Using Random Forest. Oncol. Lett. 2019, 18 (2), 1597-1606.

[18]. Liang, W.; Luo, S.; Zhao, G.; Wu, H., Predicting Hard Rock Pillar Stability Using GBDT, XGBoost, and LightGBM Algorithms. Mathematics 2020, 8 (5), 765.
[19]. Ke, G. L.; Meng, Q.; Finley, T.; Wang, T. F.; Chen, W.; Ma, W. D.; Ye, Q. W.; Liu, T. Y. In LightGBM: A Highly Efficient Gradient Boosting Decision Tree, 31st Annual Conference on Neural Information Processing Systems (NIPS), Long Beach, CA, Dec 04-09; Neural Information Processing Systems (Nips): Long Beach, CA, 2017.
[20]. Jung, Y., Multiple predicting K-fold Cross-Validation for Model Selection. J. Nonparametr. Stat. 2018, 30 (1), 197-215.
[21]. Lundberg, S. M.; Erion, G.; Chen, H.; DeGrave, A.; Prutkin, J. M.; Nair, B.; Katz, R.; Himmelfarb, J.; Bansal, N.; Lee, S. I., From Local Explanations to Global Understanding with Explainable AI for Trees. Nat. Mach. Intell. 2020, 2 (1), 56-67.
[22]. Fujimoto, K.; Kojadinovic, I.; Marichal, J.-L., Axiomatic Characterizations of Probabilistic and Cardinal-Probabilistic Interaction Indices. Games Econ. Behav. 2006, 55 (1), 72-99.
